# A Review of Wide Bandgap Semiconductors: Insights into SiC, IGZO, and Their Defect Characteristics

**DOI:** 10.3390/nano14201679

**Published:** 2024-10-19

**Authors:** Qiwei Shangguan, Yawei Lv, Changzhong Jiang

**Affiliations:** 1 School of Physics and Electronics, Hunan University, Changsha 410082, China; shangguanqiwei@hnu.edu.cn; 2 College of Materials Science and Engineering, Hunan University, Changsha 410082, China

**Keywords:** a-IGZO, 4H-SiC, defects, charge transition levels, formation energy, stability, post-process

## Abstract

Although the irreplaceable position of silicon (Si) semiconductor materials in the field of information has become a consensus, new materials continue to be sought to expand the application range of semiconductor devices. Among them, research on wide bandgap semiconductors has already achieved preliminary success, and the relevant achievements have been applied in the fields of energy conversion, display, and storage. However, similar to the history of Si, the immature material grown and device manufacturing processes at the current stage seriously hinder the popularization of wide bandgap semiconductor-based applications, and one of the crucial issues behind this is the defect problem. Here, we take amorphous indium gallium zinc oxide (a-IGZO) and 4H silicon carbide (4H-SiC) as two representatives to discuss physical/mechanical properties, electrical performance, and stability from the perspective of defects. Relevant experimental and theoretical works on defect formation, evolution, and annihilation are summarized, and the impacts on carrier transport behaviors are highlighted. State-of-the-art applications using the two materials are also briefly reviewed. This review aims to assist researchers in elucidating the complex impacts of defects on electrical behaviors of wide bandgap semiconductors, enabling them to make judgments on potential defect issues that may arise in their own processes. It aims to contribute to the effort of using various post-treatment methods to control defect behaviors and achieve the desired material and device performance.

## 1. IGZO

### 1.1. Introduction

Semiconductor materials can be broadly categorized into amorphous and crystalline types based on their structures. Amorphous semiconductors lack a well-defined crystal lattice, which grants them a greater flexibility in terms of fabrication and processing. While the electronic performance of amorphous semiconductors may not match the highly ordered crystalline counterparts, they offer significant advantages in terms of production efficiency and cost-effectiveness. As carrier mobilities in amorphous oxide semiconductors (AOS) have improved, amorphous indium-gallium-zinc (In-Ga-Zn) oxide (a-IGZO) has received considerable interest due to its fascinating physical properties [1,2]. This interest was sparked by the initial discovery in 2004 of its exceptional potential in the realm of thin film transistors (TFTs) [3], leading to substantial advancements over the subsequent two decades.

Compared with other TFT channel materials used in flat panel displays (FPDs), such as hydrogenated amorphous silicon (a-Si:H), high-temperature annealed polycrystalline silicon (HTPS), and laser-annealed low-temperature polycrystalline silicon (LTPS), a-IGZO stands out due to its superior mobility (>10 cm^2^/V·s), reduced subthreshold swing (*SS*~100 mV/dec) [4], minimal leakage current (<10^−12^ A/μm), and enhanced stability. Although high mobilities (even >100 cm^2^/V·s) are also occasionally reported in poly-Si, the presence of grain boundary (GB) issues often leads to inconsistent device performance and reliability. In contrast, the a-IGZO is a more economical and practical choice because it can be prepared in large areas at low temperatures. Meanwhile, both the quality and uniformity can be easily guaranteed by common methods, such as magnetron sputtering and atomic layer deposition (ALD). Recently, a remarkable mobility of ~70 cm^2^/V·s was obtained in a-IGZO films prepared by plasma-enhanced ALD (PEALD) [5].

At the same gate-to-source voltage (*V_gs_*) and drain-to-source voltage (*V_ds_*) conditions, the drain-to-source current (*I_ds_*) of a-IGZO TFTs is often 1 to 2 orders of magnitude higher than that of a-Si:H, which can well meet the substantial current demands of organic light emitting diode (QLED) pixels [6]. Furthermore, a-IGZO is a wide bandgap semiconductor material (>3 eV) which is transparent in visible light and shows good foldability [7]. These advantages together boost its applications in FPDs, transparent electronic devices [8], and flexible devices [9], driving the industry towards displays with higher resolutions, higher refresh rates, larger sizes, and the advent of transparent and flexible display technologies.

### 1.2. Physical Properties

**Figure 1:**
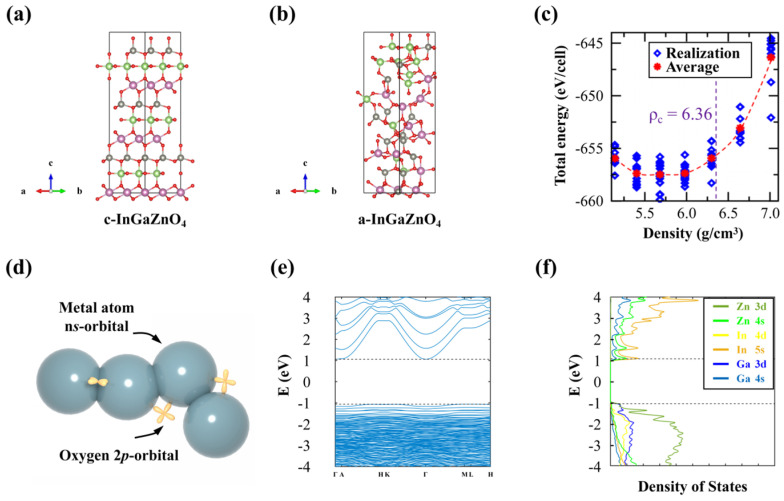
(**a**) and (**b**) Schematic illustrations of the InGaZnO_4_ in the crystal and amorphous phases. The amorphous phase is obtained by an MD-based melt-quench simulation using the crystal phase as the initio state. (**c**) Energy density curve calculated by DFT, where the diamond and star represent the energy of the individual configuration and the average energy at each density. (**d**) Schematic illustration of the real space overlaps among adjacent metal n*s* orbitals and oxygen 2*p* orbitals in AOS. The overlaps are crucial to maintain the electronic properties of AOS during the structure variation from crystal to amorphous state. (**e**) Energy band and (**f**) metal orbital DOS of an a-InGaZnO_4_ supercell containing 84 atoms generated by MD and calculated by GGA + *U* method. (**c**) Reprinted from [12], Copyright (2022) by John Wiley & Sons, Inc.

IGZO is a representative of AOS composed of three metal elements: In, Ga, and Zn. Its structures and physical properties vary significantly depending on In:Ga:Zn ratios, including the crystallinity, bandgap, and carrier concentration. A typical case is the composition with a metal ratio of In:Ga:Zn = 1:1:1 (InGaZnO_4_), whose crystalline (c-InGaZnO_4_) and amorphous (a-InGaZnO_4_) structures are shown in Figure 1a,b, respectively. The In^3+^ ions form octahedral InO_6_ units, each with six surrounding O^2−^ ions; Zn^2+^ ions form tetrahedral ZnO_4_ units, each with four surrounding O^2−^ ions; and Ga^3+^ ions form trigonal bipyramid GaO_5_ units, each with five surrounding O^2−^ ions. c-InGaZnO_4_ is composed of alternating layers of octahedral InO_6_, tetrahedral ZnO_4_, and trigonal bipyramid GaO_5_. In reality, tetrahedral Zn^2+^ ions and trigonal Ga^3+^ ions can exchange positions with each other, forming a mixed Ga/Zn layer consisting of trigonal bipyramidal units [10,11]. This positional exchange, as indicated by first-principles calculations, results in a negligible energy variation of approximately 0.1 eV, highlighting the dynamic nature and metastability of IGZO’s crystal structure.

Table 1 shows the reported bond lengths, coordination numbers, and densities of a-InGaZnO_4_ [13,14,15,16,17,18]. While discrepancies in the reported data are evident, a consistent trend emerges regarding the bond lengths: the In-O bond lengths are the longest, while the Ga-O bond lengths are the shortest, followed by the intermediate Zn-O bond lengths. This result suggests that the amorphization process in IGZO does not significantly change the bond lengths but only changes the bond angles and reduces the coordination numbers of the metal atoms. Meanwhile, the density data in Table 1 also show that the densities of a-InGaZnO_4_ could vary in a large range from 5.58 to 6.1 g/cm^3^, indicating the diversity in local a-InGaZnO_4_ structures. Indeed, as shown in Figure 1c, Medvedeva et al. [12] studied the energies of 10 a-InGaZnO_4_ structures with different densities generated by molecular dynamics (MD) simulations and found that even at the same density, the energy deviations of different structures still reached about 3 eV. Conversely, a-InGaZnO_4_, with a large density range, could also have similar energies, verifying the wide density distributions in a-InGaZnO_4_ and indicating that significant structural evolutions could occur due to a slight deposition condition variation. They also calculated the theoretical metal K-edge extended X-ray absorption fine structures (EXAFS) of a-InGaZnO_4_ at different densities and found that they matched with experimental values well, which further confirms the structural instability of IGZO.

The high electron mobility of AOS depends on the overlaps of *s* orbitals between adjacent heavy cations, as shown in Figure 1d. Hosono et al. [19] predicted that for AOS with heavy post-transition metal cations and an electronic configuration of (n − 1)*d*^10^n*s*^0^ where n ≥ 5, smaller effective electron masses and larger electron mobilities can be obtained. The subsequently discovered high-mobility AOS materials confirmed this conclusion, such as 2CdO-PbO_2_ [20], AgSbO_3_ [21], and IGZO [3]. Consistent with the orbital overlap, the conduction band minimum (CBM) of a-InGaZnO_4_ is mainly composed of spatially spreading In 5*s* orbitals with large isotropic spherical extensions in real space, leading to overlaps of *s* orbitals between adjacent metal atoms and showing insensitivity to disorders. Therefore, the field effect mobility (*μ_FE_*) of crystal-InGaO_3_(ZnO)_5_ (a kind of IGZO allotrope) is 80 cm^2^/V·s [22], while it can still maintain above 10 cm^2^/V·s in amorphous phases [23,24]. In contrast, Si atoms in a bulk state are connected to adjacent atoms by *sp*^3^ hybrid orbitals, which show a strong directional selectivity, thus leading to the electron mobility declining from 1500 cm^2^/V·s in crystal-Si (c-Si) to less than 1 cm^2^/V·s in a-Si:H [25].

As shown in Figure 1e,f, the high electron mobilities in a-IGZO can be further verified by the band structure and density of states (DOS) of a typical a-IGZO model using the density functional theory (DFT) + *U* calculation. The *U* values applied for In 4*d*, Ga 3*d*, Zn 3*d*, and O 2*p* orbitals are 7, 8, 8, and 7, respectively [26]. Figure 1f shows the valence band maximum (VBM) and CBM of the a-IGZO are mainly composed of In 5*s* and O 2*p* orbitals, respectively. The band structure of Figure 1e shows that the VBM exhibits a low dispersion, resulting in a large hole effective mass, while the CBM exhibits a high dispersion and a low electron effective mass (0.2·*m_e_* where *m_e_* is the electron mass in a vacuum), which is just slightly larger than that of the c-IGZO (0.18·*m_e_*) [14,15], indicating that the amorphous state does not severely degrade the high electron mobility in the crystal state. Note that due to the well-known problem of the bandgap underestimation in DFT calculations, the bandgaps of a-IGZO in previous reports are often about 1 eV, much smaller than the experimental values near 3.2 eV [25]. This problem can now be solved using DFT + *U* calculation to obtain a larger bandgap of 2.8 eV, similar to the experimental values.

IGZO also exhibits different physical and electrical properties due to different composition ratios of the metal atoms In:Ga:Zn, impacting their device performance. Table 2 shows the metal atomic composition ratios corresponding to the best device performance from different research groups and using different preparation processes, such as magnetron sputtering, solution processing, and PEALD [5,23,27,28,29,30,31,32]. Generally speaking, increasing the proportion of In atoms will increase the electron concentration and mobility but lead to an obvious negative shift in the turn-on voltage (*V_on_*) and the threshold voltage (*Vth*) and cause device stability issues. Increasing the proportion of Ga atoms will enlarge the IGZO bandgap and reduce the electron concentration due to the stronger ionic Ga-O bond strength (the dissociation energy of the Ga-O bond is 2.04 eV, higher than the 1.7 eV of the In-O bond and 1.52 eV of the Zn-O bond [15]), reducing the mobility but enhancing the device stability. Increasing the proportion of Zn atoms is beneficial for a-IGZO to maintain uniform amorphous phases.

**Figure 2:**
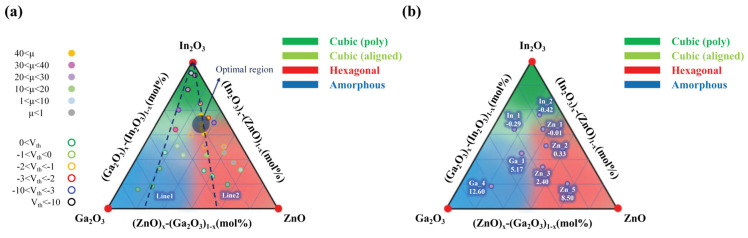
Ternary phase diagrams of IGZO fabricated by PEALD technology. The *μ_FE_*, *Vth* (**a**), and *Vth* shifts under PBTS (**b**) are also shown. (**a**,**b**) Reprinted from [32], Copyright (2023) by Wiley-VCH GmbH.

The quality of a-IGZO film also depends on the preparation technology to a great extent. Hosono et al. [19] used a pulsed laser deposition to prepare IGZO thin films and found that the films remained amorphous in a large compositional range. Hong et al. [32] used the PEALD technique to deposit IGZO films and precisely controlled the chemical compositions by adjusting the deposition cycle numbers of In_2_O_3_, Ga_2_O_3_, and ZnO layers. They obtained a larger crystallization range, as shown in Figure 2, by studying the performance of bottom-gate and top-contact IGZO TFTs. In the Zn-rich and In-rich regions, a hexagonal-ZnO (100) and a cubic-bixbyite In_2_O_3_ crystal structure appeared, as shown by the red and light green colors. As the In component increasing further, the IGZO even showed a polycrystalline structure, as shown by the dark green color. The reason may be related to the PEALD, which can provide enough reaction energy to anneal the structure during the deposition of IGZO thin films. At the same time, thick metal oxide layers with large compositional ratios are also beneficial to the growth of crystal structures.

### 1.3. Defects

Due to their excellent characteristics, such as high mobility and conductive current, low leakage current and operating voltage, and good uniformity, a-IGZO TFTs exhibit a broad application prospect in the display domain and can be used as high-performance driver devices for liquid crystal display (LCD) or OLED pixels. However, there are still many scientific problems with the commercialization of a-IGZO TFTs, and the first one is the instability issue, which seriously threatens their performance reliability. The primary cause of the instability problem is the intrinsic defects in the a-IGZO channel or channel/gate interface, which can trap or release carriers under different stress conditions. The trapped or released carriers can change the charge distribution and the band structure of the a-IGZO TFT, resulting in shifts to important properties such as *Vth*, *SS*, and current on-to-off ratio (*I_on_/I_off_*). The instability issue of a-IGZO TFTs can be classified according to four stress conditions: positive bias stress (PBS), negative bias stress (NBS), positive bias temperature stress (PBTS), and negative bias illumination stress (NBIS). Generally, PBS and PBTS will increase shallow acceptor states in the channel layer or capture electrons at the channel/gate interface, resulting in a positive shift in *Vth*, while NBS and NBIS will increase shallow donor states in the channel layer or capture holes at the channel/gate interface, resulting in a negative shift in *Vth*. Therefore, to improve the stability of a-IGZO TFT, the first target is to suppress the shallow trap state generation and the subsequent *Vth* shifts.

**Figure 3:**
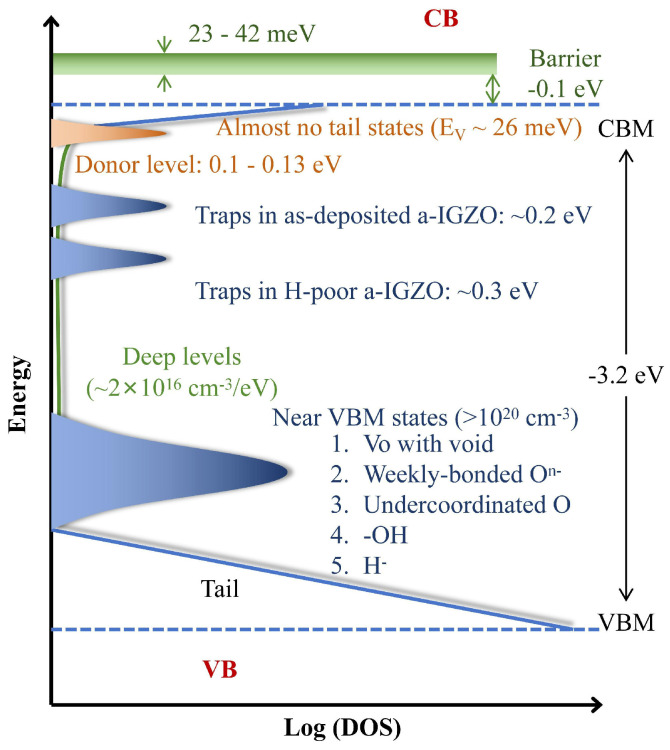
Known subgap states in the band structure of a-IGZO. Reprinted from [6], Copyright (2019) by Wiley-VCH GmbH.

Several studies have investigated the defects in a-IGZO using experimental and theoretical methods. Figure 3 summarizes the defect states of a-IGZO that have been reported so far. It can clearly be seen that the a-IGZO shallow donor level is mainly located at 0.1 to 0.13 eV below the CBM. Additionally, two deeper trap state regions at 0.2 and 0.3 eV from the CBM have also been detected from different manufacturing processes. These two state groups can be effectively eliminated or passivated by annealing at a temperature >300 °C and by hydrogen (H) doping, respectively. From the VBM to the mid-gap, a-IGZO exhibits a large trap state density and scope (~10^20^ cm^−3^ and *E_V_* + 1.5 eV), which was observed by hard X-ray photoelectron spectroscopy using a synchrotron radiation facility [33]. These states have no effect on the TFT characteristics under the normal operation mode because they are far below the Fermi level. However, they can cause degradation to the device’s performance under NBIS. Moreover, due to the existence of such a large density of trap states, the leakage currents of a-IGZO TFTs will be enlarged, and the Fermi level cannot be pushed downwards under negative *V_gs_*, impeding the opening of p-channel devices. Several hypotheses have been proposed for the origin of this density of states, such as oxygen (O) vacancies with voids, weakly bonded O, undercoordinated O, formation of -OH, and the existence of H.

#### 1.3.1. Oxygen Vacancy

An O vacancy (*V_O_*) is the most prevalent and influential point defect in a-IGZO. It is the primary source of electronic carriers. By precisely controlling the O partial pressure during the sputtering preparation process, the concentration of *V_O_* in a-IGZO can be effectively regulated [34]. In an O-deficient deposition environment, the generation of *V_O_* is favored, thereby increasing the concentration of electronic carriers and improving the conductivity of the device. Additionally, the O content in the post-annealing treatment also affects the electron concentrations of a-IGZO thin films [35]. Studies have shown that post-annealing in O_2_ or air atmospheres can lead to a reduction in *V_O_*, which in turn lowers the concentration of electronic carriers. Conversely, post-annealing in a vacuum or nitrogen (N) atmosphere can maintain the concentration while improving the positive bias stability of the corresponding devices.

**Figure 4:**
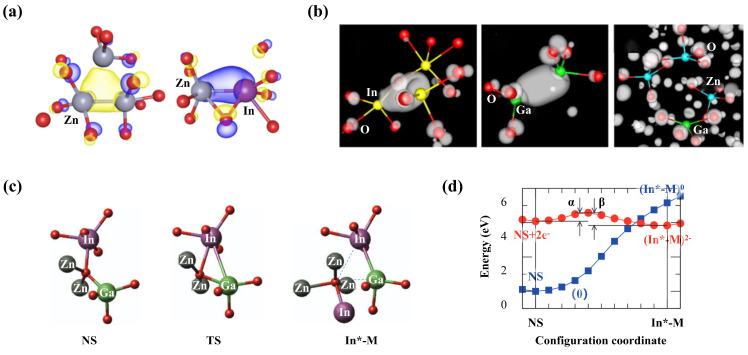
Local structure and formation energy of a-IGZO with *V_O_*. (**a**) Formation of Zn-Zn and In-Zn bonds. (**b**) Formation of In-In and Ga-Ga bonds. The *V_O_* is found to be located near Ga and Zn atoms, leading to the formation of huge voids. The In*-M bond is formed in the vicinity of the under-coordinated In (In*) by adding two electrons to the perfect a-IGZO. (**c**) Formation of the In*-M bonds and the intermediate normal state (NS) to transition state (TS). (**d**) Energy barrier diagram during the formation of the In*-M bond. (**a**) Reprinted from [38], Copyright (2018) by American Physical Society. (**b**) Reprinted from [41], Copyright (2010) by American Institute of Physics. (**c**,**d**) Reprinted from [42], Copyright (2014) by Nature Publishing Group.

To investigate the *V_O_*-induced structural evolution and the subsequent impacts on device performance, many researchers have employed DFT calculations [13,15,16,36,37,38,39,40,41,42,43,44,45,46]. To construct a realistic a-IGZO model with *V_O_*, two steps are usually involved: (1) a stoichiometric a-IGZO model is generated by the melt-quench method, which employs an MD simulation tool to simulate the rapid cooling process of a-IGZO deposition, and (2) the *V_O_* is introduced in the a-IGZO model either by directly removing O atoms or by removing O atoms and a subsequent MD melting step in order to eliminate local stresses [16]. The former preserves the local structures of *V_O_* and simulates the annealing of a stoichiometric thin film in an O-deficient atmosphere. The latter allows *V_O_* to be distributed uniformly in the model and simulates the growth of a stoichiometric thin film in an O-deficient environment. Depending on the method of introducing *V_O_*, atom number in the model, and charge state of *V_O_*, DFT calculations reveal three main microstructures of *V_O_* in a-IGZO: (1) simply forming a vacancy with dangling bonds [13,41]; (2) forming metal-metal (M-M) bonds by neighboring cations as shown in Figure 4 [13,36,38,41]; and (3) forming under-coordinated cations with reduced coordination numbers [39,42].

Using the two methods mentioned above to construct a-IGZO models containing *V_O_*, many research groups have reported the appearance and disappearance of M-M bonds, with different proportions. Noh et al. [13] found that in their model, most of the metal atoms (87.9%) around the *V_O_* would relax inward to form M-M bonds, while a small part of the metal atoms (12.1%) would relax outward to form larger voids. De Jamblinne De Meux et al. [36] obtained the opposite result in their models, as most of the cases (55%) did not form M-M bonds. They attributed this phenomenon to the exaggerated defect densities caused by the finite supercell sizes, since the formation of M-M bonds would cause large position relaxations until the atoms were far away from the defects, which already exceeded the supercell sizes. In addition, the differences among the study results may also be related to the electron concentrations of the systems. When one O atom is removed from the stoichiometric a-IGZO model, two delocalized electrons will be added to the conduction band, and the metal atoms with dangling bonds may bond with each other, forming M-M bonds, capturing the two delocalized electrons and generating localized states in the bandgap. Therefore, the charge state of the system can affect the formation of M-M bonds. For the positively charged a-IGZO system, there is almost no M-M bond. Conversely, M-M bonds may be formed (62.5%) when electrons are added to the O-deficient system [16]. Noh et al. [13] and De Jamblinne De Meux et al. [36] used amorphous structures containing 84 and 105 atoms, respectively. Thus, the model with less atoms would have a higher delocalized electron concentration after removing one O atom, which would be more beneficial to the formation of M-M bonds.

The formation of M-M bonds will affect the electronic structure of a-IGZO, especially the subgap states [15]. The dissociation and recovery of M-M bonds will also lead to the instability of the electrical properties in a-IGZO. The formation of M-M bonds will create a localized state occupied by charges in the bandgap, and the dissociation of M-M bonds will release the charges to the conduction band. Due to different research methods and atomic system sizes, the positions of subgap states formed by the M-M bonds at the bandgap reported by research groups are also different. Some groups reported subgap states in a-IGZO located between the VBM and the lower part of the bandgap [13,37,38]. However, other groups believed that M-M bonds would form subgap states near the CBM [16]. Subgap states in a wide energy range induced by M-M bonds may be possible because of amorphous IGZO structures. On the other hand, they may also be affected by the image interactions between periodic supercells in the DFT calculations, which are well known to underestimate bandgaps without DFT + *U* correction. Han et al. [39] observed that as the number of atoms in a-IGZO systems increased, the subgap states produced by M-M bonds in the DFT calculations tended to moved towards the VBM. Table 3 summarizes the positions of the subgap states reported by different groups and the calculated bandgaps of their a-IGZO model. The subgap states exhibit a wide distribution energetically, but they are generally located close to the midgap. Besides, the voids and under-coordinated cations caused by *V_O_* do not tend to form localized states in the bandgap, or they just form localized states near the CBM, acting as shallow donors.

After the formation of M-M bonds, the injection of two holes into the system can break the bonds, accompanied by significant structural relaxation. Therefore, by applying a negative gate voltage to the system, such as the NBS or NBIS, the Fermi level can be lowered to the vicinity of VBM, promoting the dissociation of the M-M bonds. Upon completion of structural relaxation following bond dissociation, researchers have suggested that M-M bonds would remain unrecovered, even if the two electrons were reinjected, since the energy after the M-M bond was broken would be lower than that of the existence of the M-M bond [38]. Then, no localized state would appear in the bandgap of a-IGZO, and the reinjected electrons would exhibit a delocalized behavior in the conduction band. However, previous studies have already proposed the possibility of M-M bond recovery. Ryu et al. [41] carried out an annealing study of *V_O_* at temperatures ranging from 200–400 °C through ab initio MD simulations. The results indicated that during the annealing process, some ionized *V_O_* can regain electrons and form M-M bonds, while others tend to diffuse towards In atoms. This means that the recovery of M-M bonds at *V_O_* positions may encounter potential barriers, which will increase alongside the decreasing of electron concentrations. This conclusion is supported by Nahm and Kim et al. [42], who studied the mechanism of M-M bond formation by injecting electrons into a perfect a-IGZO crystal cell. As shown in Figure 4d, their study showed that when two electrons are injected into the crystal, an M-M bond can be formed after crossing a 0.49 eV energy barrier, and this barrier will be further reduced along with the number of injected electrons. In the case of a-IGZO containing *V_O_*, we infer that the energy barrier can also be obviously reduced, and thus, the dissociation and recovery of M-M bonds are reversible processes.

Yao et al. [47] examined the influence of O content on the electrical properties and light sensitivity of a-IGZO TFTs. This study revealed that in O-poor environments, the *Vth* experiences a negative shift due to the increased *V_O_*. The shift is intensified when subjected to light, given that the presence of *V_O_* is associated with elevated electron densities, which in turn induces a negative *Vth* shift. In contrast, the increase in electron density boosts the M-M bond formation, which can offset the excessive electrons induced by *V_O_* and thereby alleviate the *Vth* shift. However, when exposed to light, the M-M bonds are prone to dissociation, releasing additional electrons. On the other hand, lights can also stimulate electrons from the VB to the CB. These dual actions result in a pronounced increase in electron density and a subsequent enhanced negative *Vth* shift. Furthermore, Kim et al. [48] reported a resistance switching effect in a-IGZO, and they believed that the O partial pressure during deposition played the decisive role in controlling the electrical properties of their TFTs, which was related to the bistable nature of *V_O_*.

In summary, *V_O_* plays a crucial role in a-IGZO because it is the primary source of electronic carriers, acting as donors. However, as the electron concentration increases, the formation of M-M bonds becomes possible. M-M bonds are electron capture centers, thereby compensating the extra electrons introduced by *V_O_*.

#### 1.3.2. Oxygen Interstitial

**Figure 5:**
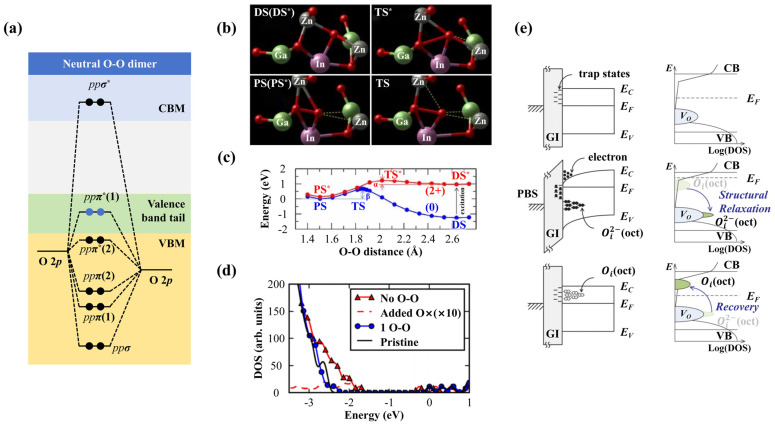
(**a**) Schematic illustration of the orbital energy levels of a neutral O-O bond. The blue circles represent the pair of electrons donated by adjacent metal ions. (**b**) Local atomic configurations of the O-O bonds formed in a-InGaZnO_4_. These configurations include the disordered state (DS), transition state (TS), and peroxide state (PS) in the neutral condition, along with their corresponding +2 charged states DS*, TS*, and PS*. (**c**) Energy barriers associated with the formation and dissociation of the O-O bond calculated by DFT in both neutral (blue curve) and charged (red curve) states. (**d**) DOS for a-InGaZnO_4_ under different conditions. The O-O bond is formed by the introduction of *O_i_*. (**e**) Band structure diagrams showing the instability caused by *O_i_* under PBS. (**a**–**d**) Reprinted from [38,49], Copyright (2015, 2018) by American Physical Society. (**b**,**c**) Reprinted from [50], Copyright (2012) by WILEY-VCH Verlag GmbH & Co. KGaA, Weinheim. (**e**) Reprinted from [51], Copyright (2017) by IEEE.

An interstitial O atom (*O_i_*) is another common defect in a-IGZO, which is introduced by foreign O atom invading. These extra O atoms can simultaneously bond to a host O atom and nearby metal atoms in a-IGZO, forming peroxide defects (O-O bonds) [49]. An invading O atom will capture two electrons, while the transformation to an O-O bond will release the electrons again, indicating that the O-O bonds are hole capture centers and can act as shallow acceptors in a-IGZO [38]. Similar to M-M bonds, *O_i_* is not essential for the O-O bonds because the bonds are also significantly affected by charge states. Simulation results have shown that the normal disorder state (without O-O bond) is stable in stoichiometric a-IGZO, while a peroxide state (O-O bond) will generate after the injection of two holes [50]. Figure 5b shows the local structure between the disorder state and peroxide state, while Figure 5c shows the energy curves for the neutral and charged states. Under the neutral charge condition, a 1.25 eV energy barrier should be crossed before forming a metastable O-O bond. After two holes are injected, the energy of the normal disorder state increases and exceeds the peroxide state by 0.88 eV, resulting in a small energy barrier of only 0.26 eV to form a stable O-O bond but a large barrier of 1.14 eV to break it. Conversely, if two electrons and *O_i_* are added to the stoichiometric a-IGZO at the same time, the relatively independent *O_i_*^2−^ defect is preferred, and an high energy barrier of 3.28 eV is needed to transform it into the O-O bond [49]. The energy barrier of breaking the O-O bond is only 0.83 eV in the electron injection condition, in good agreement with the measured values of 0.88 and 0.95 eV from as-grown and annealed samples. Besides, this bond breaking barrier also depends on the type of metal atoms nearby, among which the value may be the largest if the surrounded ions are In due to In-O having the weakest bond strength. The above analyses indicate that forming or breaking an O-O bond is decided by the charge state of the a-IGZO system to a great extent. Hole injection could induce O-O bonds, while electron injection could eliminate them, regardless of whether the system contains *O_i_* or not.

Figure 5a shows the positions of the bonding and antibonding states of the O-O bond, which are similar to those of O_2_ molecules. The bonding orbitals *ppσ*, *ppπ*(1), and *ppπ*(2) are in the deep valence band, while the antibonding orbitals ppπ*(1), ppπ*(2), and ppσ* are located near the tail of the valence band or in the conduction band. When two electrons are injected into the system, they will occupy the ppσ* state and weaken the O-O bond. As the bond is broken, the electrons will accumulate at *O_i_* and form defect states like the *p* orbital states of an isolated *O*^2−^ atom, turning the ppσ* state into a new nonbonding defect state near the valence band tail [49]. Note that this nonbonding defect state will possess lower energies when the nearby metal atoms of *O_i_* are Ga [49]. This is consistent with the X-ray photoelectron spectroscopy (XPS) experimental results, which reported that the band tail of a-IGZO increased with the O concentration [52]. The DOS plot in Figure 5d also shows similar results and indicates that the O-O bond breaking caused a more extended band tail.

From the above introduction, *O_i_* defects can act as either the electron donors or acceptors depending on the state conversion between the O-O bond and the *O_i_*^2−^ (bistable state) by applying bias stress or photoexcitation. This state conversion is usually reversible, with moderate energy barriers in between, which lead to the device’s instability. A study by Ide et al. [53] revealed that a-IGZO TFTs with excessive O atoms due to post-O_3_ annealing at 300 °C could demonstrate bistable transfer characteristics. In the absence of gate bias stress, the *SS* of the TFTs degraded relative to those annealed at lower temperatures. When a gate bias of 40 V was applied, the transfer characteristic curves all shifted positively, increasing the *Vth* and improving the *SS*. They attributed the variations to the *O_i_* being switched from an M-O weak bonding state to an M-O^−^ strong bonding state caused by the negative-*U* effect and the injected electrons. This switching pushed the *O_i_*-induced trap states downwards to the valence band tail and improved the *SS* property. In our opinion, the bistable state introduced above can also be used to explain the device performance variations. The improved *SS* remained stable for at least 12 h without light exposure, but could be reversed to the original degraded value upon rapid illumination. The photon energy needed for this recovery exceeded 2.3 eV. This phenomenon was also supported by the experimental results of relevant researchers, as shown in Figure 5e [51,54,55]. For example, Choi et al. [54] also observed PBS and NBIS instabilities at their self-aligned-top-gate coplanar IGZO TFTs fabricated in an O-rich environment. They reported an increase in the density of subgap states at *E_V_* + 1 eV by *O_i_*, consistent with the speculation of [53], but *SS* degradation was not observed. Jeong et al. [55] also noted PBS instability caused by excessive O, but they claimed that NBIS stability was reinforced, contradicting [54].

#### 1.3.3. Hydrogen Interstitial

H is the most common foreign impurity in a-IGZO thin films, and it is most likely to be introduced from the ambient H_2_O and H_2_ during the deposition process. The concentration of H impurities can be as high as 10^20^ cm^−3^, and they can exist in two different forms: positively or negatively charged H^+^ and H^−^ [56]. The ratio of H^+^ to H^−^ is about 2:1 [57], and their presence can be detected by infrared absorption spectroscopy, which shows distinct peaks for each type and obtains the corresponding concentrations from the absorption coefficients [56]. H impurities can affect the electronic properties of a-IGZO by forming different types of bonds. When they bond with O atoms, such as the ones coordinated with an In and two Zn atoms [15], they form a hydroxyl group (O-H) and become H^+^, acting as a donor impurity and increasing the electron concentration and conductivity of a-IGZO [15,58]. This reaction is favored in the O-rich environment [59]. On the other hand, H can also bond with metal atoms to form M-H bonds and become H^−^. This requires either doping H into *V_O_* or having negative charges in the system [38]. Generally, in a system with multiple H impurities, some of them may form O-H bonds, while others may form M-H bonds consistent with the experimental observation of both H^+^ and H^−^ states in a-IGZO, but more studies should be carried out to further understand their roles in device performance.

**Figure 6:**
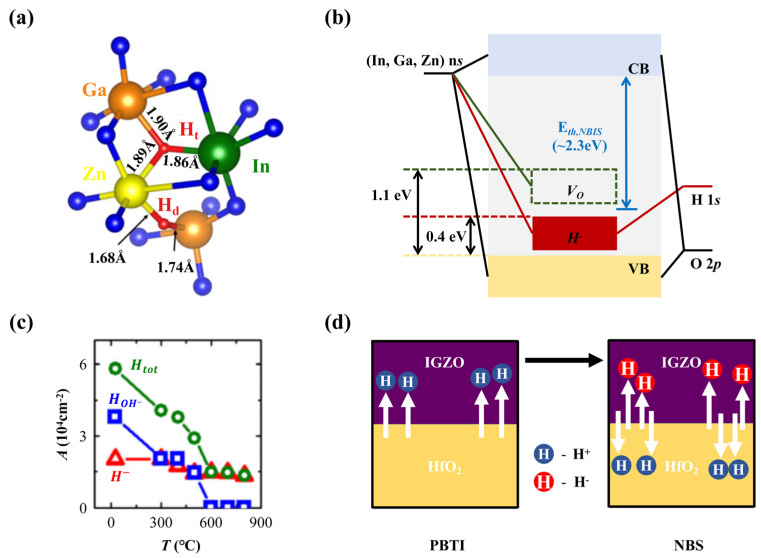
(**a**,**b**) Schematic diagram of the local a-IGZO structure with H impurities located at the *V_O_* positions and induced subgap states. H_t_ and H_d_ in (**a**) refer to H atoms that are triple-coordinated and double-coordinated. The blue arrow in (**b**) denotes the threshold photon energy (*Eth*, NBIS) associated with the NBIS phenomenon. (**c**) Schematic illustration of the integrated peak areas corresponding to the M-H and O-H vibrational modes in a-IGZO films, as determined by the TDS. (**d**) Diagram of H distributions in a-IGZO TFTs during PBTI and NBS. During PBTI, H^+^ are formed in HfO_2_ and moved to the channel, increasing carrier densities. Under NBS, H^+^ returns to HfO_2_ or changes to H^−^ in the channel. Both H states lead to negative Δ*V_th_*. (**a**–**c**) Reprinted from [56], Copyright (2017) by AIP Publishing. (**d**) Reprinted from [63], Copyright (2024) by IEEE.

Usually, bonding with H atoms can reduce system energies. Therefore, if there is no defect level in the bandgap before H doping, the formation of O-H or M-H bonds will not create subgap states, except for some accidental band tail states near 0.4 eV above the VBM generated by the formation of O-H bonds, which cause the deformation of O-M bonds [60,61]. If there is a defect level in the bandgap, such as the *V_O_*-induced In-Zn defect at 1.1 eV above the VBM, the formation of M-H bonds will lower the defect level to 0.4 eV above the VBM, as shown in Figure 6a,b [56]. Moreover, H is able to reduce the diffusion barrier of O in experiments, which is beneficial to *V_O_* repairing during the annealing process in an O_2_ atmosphere [62]. To some extent, H impurities can be used to restrain the trap effects induced by other defects in a-IGZO.

Intuitively, O-H bonds should be more stable than M-H bonds. However, the opposite has been found in experiments. As stated above, the formation of an M-H bond requires the presence of at least one free electron or *V_O_* in the a-IGZO model. When one free electron is assumed, the O-H bond will possess an energy which is 0.7 eV lower than the M-H bond, but the M-H to O-H transformation barrier is 1.63 eV [38]. When the M-H is assumed to be generated at a *V_O_* position, a barrier of 1.85 eV should be crossed to move the H atom to the nearest O atoms and produce an O-H bond [43]. In both assumptions, the barriers to break an M-H bond are high. Conversely, the barrier of a H atom migrating from an O site to another O site nearby is only 0.16 eV, implying that the diffusion of H atoms through O positions is much easier than through metal atom positions [64]. Noh et al. [65] obtained similar results with a dissociation energy of an M-H bond of 1.27 eV and a migration barrier of an O-H bond of 0.51 eV. This speculation can be verified by the infrared absorption spectra of the two bond types [56]. From the absorption peak calculations, the concentration variations of O-H and M-H bonds along annealing temperature is plotted in Figure 6c. When the temperature exceeds 600 °C, most of the O-H bonds are broken, while the M-H defects remain at a relatively high concentration.

The bistable behaviors of H atoms are often observed in metal oxide semiconductors [66]. In a-IGZO, the presence of O-H and M-H bonds can generate bistable behaviors that vary with the electron density and *V_gs_*. At low *V_O_* and H concentrations, H atoms predominantly exhibit the H^+^ state [67], resulting in the formation of O-H bonds, thereby serving as donors and enhancing the material’s electrical conductivity. Reversely, at high *V_O_* and H concentrations, H atoms are more likely to be found in the H⁻ state, either filling *V_O_* sites or forming M-H bonds with neighboring metal atoms [47]. This process in turn serves to neutralize excessive electrons. Liu et al. [63] further assessed the stress and recovery performance of a-IGZO FETs by considering the oxide layers as H impurity donors and acceptors. As shown in Figure 6d, they verified the presence of H impurities in dual charge states, and in both states, they could induce negative *Vth* shifts and increase channel carrier concentrations [63,68]. Moreover, H concentrations also influence the structures of a-IGZO, as films with insufficient H impurities generate significant structural stresses, reducing the structural density and causing instability issues [69]. Therefore, proper H levels are indispensable to optimize the a-IGZO matrix [69]. However, an excessive H concentration can increase the electron density, leading to a negative shift in the *Vth* and impacting device stability during the process of H desorption [70].

In summary, H impurities can be used to passivate the defect-induced trap states in a-IGZO bandgaps and release strains. Their most stable position is *V_O_*, and they can be eliminated by migrations via O atoms in the annealing process.

#### 1.3.4. Defects’ Impacts on Device Performance

Defects can significantly impact a-IGZO device performance, affecting parameters such as carrier mobility, *Vth*, *SS*, and overall stability. Next, these properties are discussed in the presence of various stress conditions.

**Figure 7:**
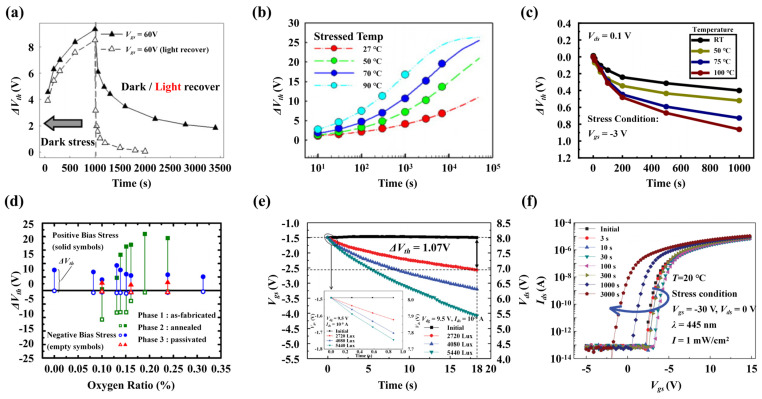
Stability issue of a-IGZO TFTs. (**a**) *Vth* recovery process in dark and light environments. (**b**,**c**) *Vth* variations under PBS and NBS. (**d**) *Vth* variations along O ratio under PBS and NBS. (**e**) *V_gs_* variations under NBIS. The inset shows the time response within 1 s. (**f**) Transfer characteristic curves of a-IGZO TFTs under NBIS. Notably, the *Vth* exhibits a two-stage degradation pattern. Initially, there is a positive shift, followed by a negative shift as the stress continues to increase. (**a**,**b**) Reprinted from [71,73], Copyright (2010 and 2011) by AIP Publishing. (**c**–**f**) Reprinted from [75], [74,76,77], Copyright (2011, 2018, 2019, and 2024) by IEEE.

The positive bias stress instability (PBS instability) in a-IGZO TFTs manifests as a discernible shift in the transfer characteristic curve accompanied by an extended recovery time after the stress. In detail, the *Vth* experiences a positive shift, while the *SS* usually remains unaffected [71]. The PBS instability is notably influenced by illumination, and exposure to light during the PBS can obviously relieve the *Vth* increase. For an example, Toledo et al. [72] found that once a positive Δ*V_th_* was established, it might require up to 24 h to recover the initial state in the absence of light, while the recovery time was drastically reduced to approximately 2 h with illumination. As shown in Figure 7a, a similar device recovery behavior was reported in [71]. The underlying cause of PBS instability in a-IGZO TFTs is believed to stem from electron trapping at the channel, oxide layer, or their interface. By empirical fitting of the stretched-exponential equation describing the *Vth* shift, Chen et al. [71] revealed that the potential barrier for electron trapping was approximately 0.38 eV, and for electron excitation during the *Vth* recovery phase, it was around 0.23 eV. This small recovery barrier elucidates that the trapped electrons can be released by illumination. Indeed, illumination serves to excite electrons, shifting the transfer characteristic curve negatively, even in the absence of a gate bias, thereby counteracting the positive *Vth* shift induced by the PBS [71,72]. Another investigation identified larger values for the formation and recovery barriers of the PBS instability of 0.95 and 0.97 eV [73]. From the studies of these energy barriers, the PBS instability is associated with the genesis and migration of defects, such as the *V_O_*. As the temperature increases, the PBS instability in a-IGZO TFTs is aggravated, which is clearly shown in Figure 7b, although the *Vth* recovery is also accelerated [73]. It has been observed that under identical PBS conditions, the a-IGZO film with a higher O content displays a more serious *Vth* shift and *SS* degradation [51,53]. In Figure 7d, the as-fabricated a-IGZO demonstrates a performance degradation trend under PBS, which intensifies with the increase in the O ratio. However, once the internal defects are reduced through annealing and passivation processes, there is a significant improvement in the *Vth* drift under PBS conditions [74]. Collectively, these findings suggest that the PBS instability in a-IGZO TFTs is likely correlated with both the *V_O_* and *O_i_* defects.

In a-IGZO TFTs, the negative bias stress instability (NBS instability) is a negative shift phenomenon in the transfer characteristic curves when negative bias stresses are applied, and the shift becomes larger as the temperature increases, as shown in the Figure 7c [75]. This instability can typically be related to the hole traps in the gate oxide layer, the a-IGZO film, and their interface. Studies have shown that with an increase in O content, the NBS stability of post-deposition a-IGZO films can be significantly improved, while the corresponding PBS instability is aggravated [74]. The improvement can be explained by the passivation of *V_O_* in the a-IGZO channel. When negative *V_g_* is applied, the M-M bonds due to V_O_ defects become unstable as the Fermi level moves downward, until they break and form ionized *V_O_*^2+^ defects. These *V_O_*^2+^ defects accumulate at the oxide/a-IGZO interface, leading to the NBS instability of a-IGZO TFTs. Additionally, the capture of holes by *O_i_* to form O-O bonds may also be a source of the instability, but it can be ruled out due to the experimentally reported fast *Vth* recovery time on the same order of the stressing time. If the *Vth* shift is caused by the formation of O-O bonds, it is impossible to recovery so quickly [38].

In a-IGZO TFTs, negative bias illumination stress instability (NBIS instability) is another commonly discussed problem, especially when used in display backplane driving circuits, since the light of display pixels can be naturally regarded as the illumination stress. Figure 7e illustrates the more serious deterioration in NBIS stability in a-IGZO TFTs as a function of increasing light intensity and prolonged illumination time [76]. High-quality films immune to NBS instability can still exhibit negative shifts in transfer characteristic curves after illumination [70]. Mativenga et al. [78] showed that NBIS instability was closely related to photo-generated holes. They found that under illumination, the concentration of *V_O_* in the a-IGZO back channel significantly increased, while the number of M-O bonds decreased. The increase contributes additional electrons, which should be the root cause of the negative *Vth* shift. A study by Ryu et al. [41] also supported this view, suggesting that NBIS instability is related to *V_O_*. Under the combined effects of NBIS, the electrons and holes generated by illumination will drift away from and towards the gate oxide/a-IGZO interface, respectively. When holes move to the interface, they are captured by nearby *V_O_*, forming *V_O_*^2+^. Once the NBIS is removed, due to the accumulated *V_O_*^2+^ near the interface, the energy bands between a-IGZO and the oxide will bend, forming a potential well and maintaining the negative shift in *Vth* [41,79]. Choi et al. [54] presented an alternative perspective. They declared that while *V_O_* contributes to NBIS instability, the primary cause is attributed to O-O bonds formed by excessive O. These bonds induce a negative *Vth* shift, as demonstrated in the subsequent reaction equation:

(1) $$\begin{array}{c} O^{2-}+O_{ex}^{2-}+hv\to {\left(O-O_{ex}\right)}^{2-}+2e^{-} \end{array}$$

To improve the device’s NBIS stability, appropriate H impurities can be added into the film. They will form M-H bonds at the *V_O_* sites to passivate the defects [65]. However, excessive H impurities should also be avoided, since they will be dissociated from a-IGZO gradually and cause poor thermal stability [70]. Furthermore, as illustrated in Figure 7f, Li et al. [77] observed a two-stage degradation behavior in a-IGZO TFTs under NBIS conditions. Specifically, there was an initial positive shift in *Vth*, followed by a subsequent negative shift. The initial positive *Vth* shift is believed to be induced by the residual water present at the oxide interface of the a-IGZO TFTs channel.

#### 1.3.5. Post-Processes

To relieve the performance degradation and instability in a-IGZO TFTs arising from inherent defects, researchers have innovated many post-fabrication treatments, including thermal annealing, plasma enhancement, and other advanced techniques. The strategic application of these methods markedly improves the stability and extends the durability of a-IGZO TFTs, paving the way for their applications in future electronics, such as superior-quality displays, dynamic random-access memory (DRAM), and sensors.

**Figure 8:**
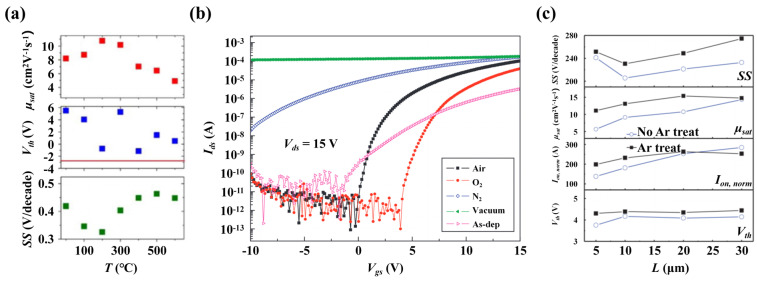
(**a**) Impacts of dry-O annealing at various temperatures on the *SS*, *μ_sat_*, and *Vth* of a-IGZO TFTs. (**b**) Transfer characteristics of both the as-deposited TFT and a-IGZO TFTs post-annealed in various environments. (**c**) *SS*, *μ_sat_*, normalized current (*I_on, norm_*), and *Vth* of a-IGZO TFTs with and without Ar plasma treatment along channel lengths (*L*). (**a**) Reprinted from [83], Copyright (2013) by AIP Publishing. (**b**) Reprinted from [86], Copyright (2011) by American Scientific Publishers. (**c**) Reprinted from [87], Copyright (2016) by IEEE.

The annealing temperature is a key parameter to promote the performance of a-IGZO TFTs. Kim et al. [80] reported that annealing at low temperatures, such as 120 °C for 2 h in an air atmosphere, could effectively reduce the trap state density and remarkably improve the *SS* property from 1.613 to 0.449 V/dec. The annealing temperature was further elevated to 300 °C by Jallorina et al. [81], and they found that the device performance was substantially boosted compared with annealing at 150 °C. The electron mobility increased from 2.36 to 13.4 cm^2^/V·s, and the *SS* reduced to 0.11 V/dec. The observations from Kikuchi et al. [82] agreed with these findings, and they also pointed out that dry-O annealing at temperatures above 150 °C could obviously enhance the performance of sputter-deposited a-IGZO TFTs. Hanyu et al. [83] further emphasized the critical role of annealing temperature and indicated that dry-O annealing within the range of 250–300 °C yielded the optimal device performance, with the highest saturation mobility (*μ_sat_)* and a *Vth* approaching 0 V, as shown in Figure 8a. However, it should be noted that exceeding an annealing temperature of 300 °C can lead to a decline in device performance. This deterioration becomes more pronounced above 600 °C, where the crystallization of a-IGZO can induce GB complications, as supported by Shin et al. [84]. The performance degradation observed at annealing temperatures between 400–500 °C may be attributed to the desorption of H_2_O, since thermal desorption spectroscopy (TDS) revealed that the desorption threshold temperature for H_2_O is 400 °C, a temperature coinciding with the onset of performance decline. This suggests that annealing at elevated temperatures can result in H depletion in a-IGZO, potentially creating new electron traps. Additionally, Choi et al. [85] indicated that for a-IGZO TFTs employing titanium (Ti) as the source/drain electrodes, annealing temperatures exceeding 300 °C could cause Ti diffusion into the a-IGZO layer, leading to the formation of TiO_x_ and a consequent device performance degradation. In summary, we believe that thermal oxygen annealing at less than 300 °C should be an effective post-fabrication treatment to improve the electrical performance and stability of a-IGZO TFTs.

The choice of annealing atmosphere also significantly influences the performance of a-IGZO TFTs. Investigations into the impacts of different annealing atmospheres on the performance of a-IGZO TFTs, including conditions like dry-O, wet-O, O_3_, N_2_, H_2_, ambient air, and a vacuum, has revealed valuable insights. Huang et al. [88] observed that annealing at 350 °C within N_2_ and H_2_/N_2_ mixtures could improve the *Vth* and *SS* behavior but reduce the *μ_sat_*, as verified by the emergence of an additional InO_x_ peak in XPS analyses. Conversely, Park et al. [86] reported that annealing in N_2_ and vacuum environments might increase *V_O_*, leading to an increased electron concentration and compromised switching behavior, while O_2_ and air environments might be beneficial to the device’s stability. The transfer characteristic curves of a-IGZO TFTs annealed under different atmospheres are shown in Figure 8b. Mudgal et al. [89] highlighted the advantage of annealing in N_2_ at 400 °C. Notably, wet-O annealing outperformed dry-O annealing in promoting the device performance, despite a potential negative shift in the transfer characteristic curve [82]. Ide et al. [90] compared O_2_ and O_3_ annealing, concluding that low-temperature O_3_ annealing at 150 °C is a better choice, likely owing to the enhanced oxidizing capacity and greater diffusion constant of O atoms from O_3_. However, they also found potential drawbacks, such as the device’s PBS instability following O_3_ annealing at 300 °C, possibly induced by excessive O-related defects [53]. Given the high *V_O_* and H impurity concentrations in a-IGZO TFTs, a comprehensive evaluation of the annealing atmosphere is imperative to optimize the annealing process.

Another research direction is annealing step modulation. Peng et al. [91] demonstrated that a two-step annealing approach, consisting of pre-annealing at 200 °C for 1 h in air following the deposition of the a-IGZO channel and post-annealing at 300 °C for 1 h after the preparation of a passivation layer, greatly improved the *SS* and stability of a-IGZO TFTs. Similarly, Jeon et al. [92] employed a two-step annealing technique to reduce the *Vth* shift from 3.7 V to 1.3 V by controlling the O-H bonds at the a-IGZO/SiO_2_ interface. Their method involved pre-annealing at 300 °C in an N_2_ atmosphere for 1 h, followed by prolonged vacuum post-annealing at 250 °C for 10 h. The pre-annealing increased the O-H bonds, thereby increasing the electron concentration and inducing a negative *Vth* shift. The post-annealing allowed for H diffusion, which improved the device’s stability. Moreover, microwave annealing (MWA) facilitates direct energy transfer to materials via polar molecule vibrations, saving time and energy in the heating process. Pi et al. [93] investigated the impact of MWA on a-IGZO TFTs and found that a-IGZO TFTs with MWA exhibited an improvement of 57% in the *SS* while maintaining a high field-effect mobility of up to 29.2 cm^2^/V·s and a large switching ratio >10^8^.

In addition to annealing, plasma treatment is also an effective way to improve the electrical performance and stability of a-IGZO TFTs, including O, H, Argon (Ar), N_2_O, CF_4_, and CHF_3_ plasmas. For example, O plasma treatment at 60 W, as demonstrated by Lee et al. [94], yielded a *Vth* of 0.4 V, electron mobility of 14.8 cm^2^/V·s, *I_on_/I_off_* of 4.8 × 10^8^, and *SS* of 0.6 V/dec. Similarly, Abliz et al. [95] found that a simple H plasma treatment could improve the electrical performance and reliability of a-IGZO TFTs by increasing the carrier concentration and reducing the surface or interface defects. On the one hand, Ar plasma treatment can weaken and break the M-O bonds near the surface of a-IGZO films, which is beneficial to the diffusion of metallic impurities such as Ti to form TiO_x_ and increase the *V_O_* concentration [87]. This increase can be compensated by the energy release of Ar ion bombardment, which repairs the ionic bonds and reduces the *V_O_* concentration in the bulk. As depicted in Figure 8c, the application of Ar plasma treatment demonstrates a promising trend towards device miniaturization. For devices of smaller channel lengths (*L*), the *SS* for both those treated and not treated with Ar plasma are similar. However, devices subjected to Ar plasma treatment exhibit a higher *μ_sat_* and normalized current (*I_on, norm_*). Although N_2_O plasma treatment can reduce the *V_O_* in a-IGZO, it may also damage M-O bonds. It is suggested that maintaining an N_2_O atmosphere after treatment can prevent broken M-O bonds and device performance degradation [96]. CF_4_ plasma treatment, according to Wang et al. [97], can remove impurities introduced during H_2_O_2_ etching and Mo electrode deposition, improving PBS and NBS stabilities. Lastly, Huang et al. [98] showed that CHF_3_ plasma treatment could prevent moisture absorption and enhance device stability.

### 1.4. Application

#### 1.4.1. Display Devices

**Figure 9:**
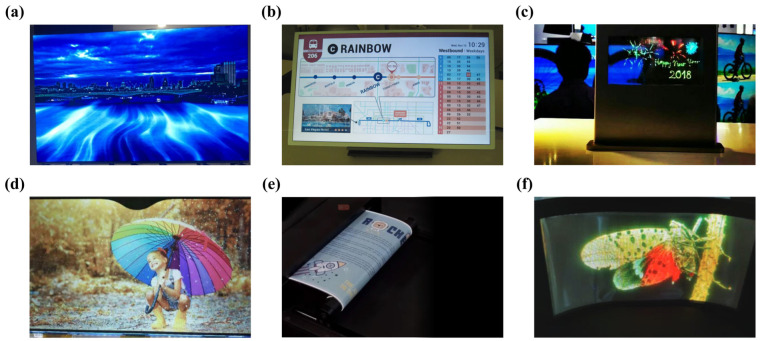
(**a**) An AM mini-LED tiled display utilizing IGZO TFT glass backplanes. (**b**) A 31.5-inch transflective LCD with a twisted vertically aligned mode. (**c**) An 8-inch transparent AM micro-LED display. (**d**) An 11.6-inch and 144 Hz LCD module with IGZO TFTs using a back channel etch structure. (**e**) An 8-inch rollable E Ink Gallery 3 color ePaper. (**f**) An 8-inch AM mini-LED display. (**a**–**f**) Reprinted from [99,100,101,102,103,104], Copyright (2019–2021 and 2023) by IEEE.

The amorphous nature of a-IGZO allows for the fabrication of devices on flexible substrates, opening new possibilities for the development of bendable and foldable displays as well as other innovative electronic applications. Moreover, the material exhibits excellent uniformity across large areas, which is crucial to produce high-resolution and large-area display electronics. Figure 9 illustrates the various applications of IGZO reported in recent years in the fields of flat panel, flexible, and transparent displays [99,100,101,102,103,104].

Since the first preparation of a-IGZO TFTs in 2004, a-IGZO has rapidly become a promising material to replace the traditional a-Si:H and poly-Si used in the display field due to its high mobility, low leakage current, good uniformity, and good stability, and thus has received extensive attention [3]. a-IGZO TFTs are especially suitable for the backplane channel of OLEDs because OLEDs require large drive currents, which is one of the main advantages of a-IGZO. In addition, a-IGZO TFTs are immune to grain boundaries and substrate bending due to their amorphous structure, which can be used in emerging flexible and transparent electronics with large-area, high-resolution, and high-uniformity merits.

Since the preparation of a-IGZO TFTs is compatible with the traditional a-Si:H deposition process, it only took 8 years for Sharp Corporation (Tokyo, Japan) to launch the world’s first smartphone Aquos Phone Zeta SH-02E with a 4.9-inch IGZO-based screen in 2012. Subsequently, Sharp developed and optimized the IGZO technology and applied it to multiple fields such as tablets, monitors, electronic paper, etc. Recently, it launched the fifth generation of IGZO technology, which promotes electron mobility by 1.5 times compared with the previous generation and further reduces power consumption. In November 2023, the latest color electronic paper based on IGZO backplane technology was jointly developed by E ink Yuan Tai Technology (Taipei, Taiwan) and Sharp. It exhibits rich and saturated colors and contrast, comparable to that of advanced color printing papers. Other companies with commercial products related to IGZO technology include LG (Seoul, Republic of Korea), Samsung (Suwon, Republic of Korea), Hitachi (Tokyo, Japan), Uda Optical (Kuala Lumpur, Malaysia), Apple (Cupertino, CA, USA), etc. In 2013, LG commercialized a 55-inch 1080-resolution OLED TV based on IGZO. In 2016 and 2017, it further commercialized the IGZO technology-based 65-inch and 77-inch 4K-resolution OLED TVs and ultra-thin Wallpaper OLED TVs.

In addition, due to the wide bandgap and moderate manufacturing temperature of a-IGZO, it also has broad application potential in the field of transparent and flexible electronics. Fan et al. [101] demonstrated an 8-inch full-color Active Matrix (AM) mini-LED transparent display with an a-IGZO TFT backplane, which has a transmittance of more than 60% and uses RGB inverted chip mini-LEDs to achieve more than 114% of the NTSC color gamut. As early as the first preparation, the flexibility of a-IGZO was demonstrated [3]. Since then, several groups have reported the preparation of flexible display screens based on IGZO [105]. In the field of wearable electronic devices, a-IGZO TFTs are also expected to be widely used in health detection, medical diagnosis, and other aspects [9]. According to statistics from Mordor Intelligence, the IGZO market value is estimated to be 2.62 billion dollars in 2024 and is expected to reach 4.54 billion dollars by 2029, with an average annual growth rate of 11.61%, showing a broad market prospect.

#### 1.4.2. DRAM

**Figure 10:**
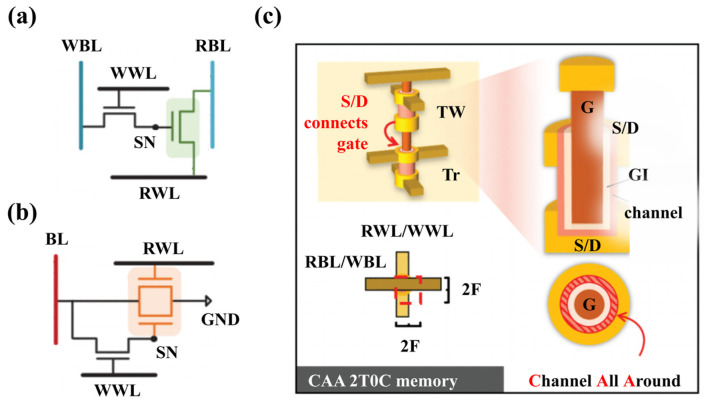
(**a**) Single and (**b**) double gate 2T0C DRAM circuits. (**c**) Bit-cell structure constructed by the IGZO-based 4F^2^ 2T0C CAA FET. (**a**,**b**) Reprinted from [111], Copyright (2022) by IEEE. (**c**) Reprinted from [108], Copyright (2022) by IEEE.

The “memory wall” is a major challenge in the current computing field, which refers to the fact that the processor performs calculations much faster than the data can be read by separate DRAM memory chips. This speed difference severely degrades the performance of AI applications, which need to process large amounts of data that often cannot be fully stored in the processor’s on-board memory, such as for facial recognition, speech understanding, and product recommendation. To reduce the data transfer time, 3D integration technology is an effective solution, which can stack DRAM memories closely together with processors. However, as the DRAM size continues to shrink, maintaining the 64 ms refresh time becomes a more difficult task, which also limits its further development. Therefore, finding a new DRAM memory structure that can achieve high-density 3D integration, improve the retention time, and reduce the power consumption has become a hot research topic. Among these studies, IGZO TFTs have received much attention due to their low off-state current, high mobility, and low temperature manufacturing process. In 2019, researchers first proposed a 2T1C (two transistors and one capacitor) DRAM cell based on IGZO TFTs to achieve a retention time of more than 10^4^ s [106]. In 2020, researchers further proposed a 2T0C DRAM cell based on IGZO TFTs using the gate oxides of two transistors as capacitors to store data, achieving a retention time of more than 400 s [107], inspiring research enthusiasm on no-capacitor DRAM cell technology based on IGZO TFTs. The bit-cell circuit diagram of a 2T0C DRAM with a single gate is shown in Figure 10a. After that, numerous research studies on the 2T0C DRAM have been published [108,109,110,111,112,113,114].

Liu et al. innovatively fabricated stackable vertical Channel-All-Around (CAA) IGZO field-effect transistors (FETs) by a back-end-of-line compatible process and the PEALD technique for the first time in 2021, as shown in Figure 10c. This technique is expected to achieve high-density 4F^2^ IGZO 2T0C DRAM cells and to have good thermal stability in the temperature range of −40 to 120 °C [108,110]. Following this idea, Lu et al. [111] proposed a novel 2T0C DRAM structure based on double-gate IGZO transistors for the first time in 2022, as shown in Figure 10b. As the key device in memory cells, read control operations and data storage are accomplished separately by the two gates. There are three obvious advantages of this DRAM cell design: (1) it reduces the number of bit lines and simplifies the circuit design and layout; (2) it avoids the IR drop problem of the read word line and improves the reliability and stability of the cell; and (3) it facilitates the construction of large-scale arrays and enhances the capacity and performance of the memory. Table 4 summarizes the performance of 2T0C DRAMs base on IGZO transistors reported in recent years. These technological breakthroughs demonstrate the huge application prospect of IGZO in the storage field.

## 2. SiC

### 2.1. Introduction

Semiconductors with strictly ordered crystal structures, such as silicon carbide (SiC), exhibit advantages in carrier mobility, thermal conductivity, and high-temperature stability due to their highly regular atomic arrangements and periodic lattice structures. Among wide bandgap semiconductors, SiC stands out as a leading material, offering exceptional potential across various industries thanks to its unique physical properties, which contribute to a high breakdown electric field, excellent thermal conductivity, superior carrier mobility, and exceptional radiation resistance.

Compared to gallium nitride (GaN), which possesses a high electron mobility and a distinct advantage in high-frequency, low-power applications [115], SiC succeeds in electric field breakdown, enabling it to withstand higher voltages—a critical feature for power electronics. Additionally, SiC’s superior thermal conductivity enhances heat dissipations in high-power devices, ensuring reliable performance even under demanding conditions. Furthermore, SiC’s unique defect structures present considerable promise in the realm of quantum computing. These defects can function as qubits, essential for the advancement of quantum devices, thereby broadening SiC’s application horizons beyond conventional electronics.

In comparison, the development of ultra-wide bandgap semiconductors like gallium oxide (Ga₂O₃) and aluminum nitride (AlN), which possess bandgaps of 4.9 eV and 6.2 eV and offer superior power densities and lower energy losses, is significantly hindered by a lack of effective p-type doping [116,117,118]. This limitation confines their commercial applications. On the other hand, SiC has already demonstrated substantial advantages in practical, large-scale preparations. SiC-based power semiconductor technology is increasingly emerging as a foundational pillar in key sectors such as electric vehicles, fast-charging infrastructure, telecommunications base stations, data center power systems, ultra-high voltage transmission networks, and rail transit systems. These advancements highlight SiC’s pivotal role in the future of semiconductor innovation, underscoring its growing significance within the ever-evolving electronics industry.

### 2.2. Physical Properties

**Figure 11:**
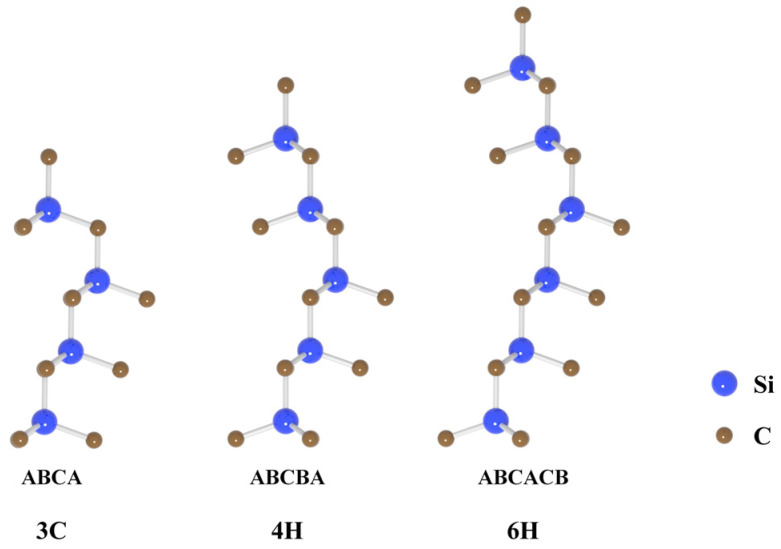
Stacking sequences of 3C-, 4H-, and 6H-SiC.

SiC is a semiconductor material composed of silicon (Si) and carbon (C) atoms. Each atom possesses four valence electrons and generates covalent bonds with four neighboring atoms via *sp*^3^; hybridization to form a tetrahedral lattice. SiC is notable for its extensive polymorphs, which include more than 250 distinct variants. These polymorphs emerge from the stacking sequences of atomic layers, with common forms being 2H-SiC, 3C-SiC, 4H-SiC, 6H-SiC, 15R-SiC, and 21R-SiC [119,120,121,122,123]. According to Ramsdell’s notation, polymorphs are labeled as nX, where ‘n’ indicates the count of SiC bilayers in the stack and ‘X’ signifies the Bravais lattice type—cubic (C), hexagonal (H), or rhombohedral (R). For example, 4H-SiC is a hexagonal SiC composed of a repeated stacking order of ABCB. Typically, 3C-SiC is referred to as β-SiC and is widely used in nanomaterials [124], while others are referred to as α-SiC [123]. The stacking sequences of the 3C-, 4H-, and 6H-SiC are shown in Figure 11. In general, crystals with strong covalent bonds crystallize into sphalerite structures, while crystals with strong ionic bonds are more stable with wurtzite structures. The polytypic phenomenon of SiC may be related to its characteristic of intermediate ionic bond [125].

The stacking variations in SiC result in lattice site disparities. Sites surrounded by a hexagonal arrangement are identified as hexagonal lattice points, and those within a cubic arrangement are known as cubic lattice points, marked with ‘*h*’ and ‘*k*’, respectively. The impact of doping and point defects varies depending on their position within the lattice structure.

The thermal stability of SiC polymorphs and their tendency for crystal nucleation are temperature-dependent [126]. High temperatures destabilize 3C-SiC and 2H-SiC and complicate the growth of large crystals. Research indicates that during vapor phase growth, 3C-SiC undergoes a transformation into the 6H-SiC crystal structure at temperatures exceeding 1900 °C [127]. Theoretical calculations also show that 4H-SiC is more stable than 3C-SiC without considering the temperature effect [128], and this conclusion can be overturned only at the Si-terminated surface condition. Therefore, 4H-SiC and 6H-SiC are more prevalent and widely researched. SiC polymorphs share similar mechanical properties, such as a Mohs hardness of roughly 9 and a Young’s modulus ranging from 330 GPa to 700 GPa, depending on the measurement technique used [129,130].

**Figure 12:**
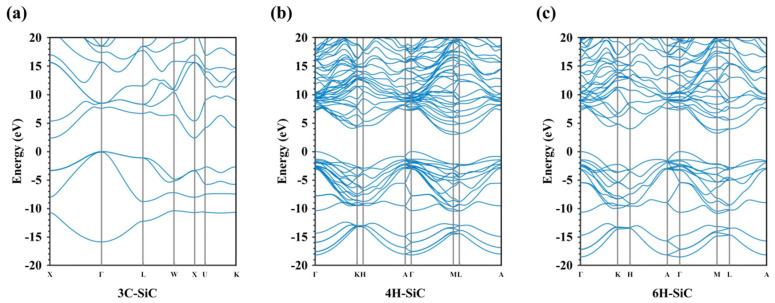
Band structure of (**a**) 3C-SiC, (**b**) 4H-SiC, and (**c**) 6H-SiC.

Despite the similar mechanical properties, SiC polymorphs exhibit distinct electronic band structures, leading to unique optical and electrical properties [119,123,131]. The experimental bandgap values of 3C-SiC, 4H-SiC, and 6H-SiC are 2.4, 3.29, and 3.1 eV, respectively [122]. Figure 12 illustrates the computed band diagrams utilizing the first-principles DFT method with a hybrid functional correction to overcome the bandgap underestimation. It can be observed that these SiC polytypes all exhibit indirect bandgaps. VBM is consistently situated at the Γ point, while CBM is located at the X point in 3C-SiC and M point in 4H-SiC and 6H-SiC.

From the above analyses, 4H-SiC stands out due to its excellent mechanical and physical properties for high-performance electrical applications. It has a high electron mobility, which allows for faster switching speeds and improved efficiency in electronic devices. This is crucial for high-frequency and high-power applications. Additionally, it also exhibits a high breakdown voltage, making it an ideal material for power electronics. Moreover, 4H-SiC is less prone to leakage currents at high temperatures due to its wide bandgap, which is essential for power devices that operate under extreme conditions. Finally, 4-inch to 6-inch 4H-SiC wafers have been mass-produced, and small-scale production of 8-inch wafers started in 2021, demonstrating the commercial viability and scalability of this material for industrial applications [132].

### 2.3. Defects

The material quality and device performance of 4H-SiC are severely affected by its internal micro-defects. Therefore, it is crucial to investigate the formation mechanisms, identification techniques, and impacts on electrical performance of micro-defects in order to enhance the quality and functionality of 4H-SiC materials and devices. The defects in 4H-SiC mainly include two categories: intrinsic defects and impurities. Intrinsic defects refer to the defects caused by the incompleteness of the lattice structure, such as vacancies, interstitials, antisites, dislocations, etc. This article mainly focuses on point defects and does not involve dislocations. Intrinsic defects can be generated in the growth, ion implantation, irradiation, and other processes of 4H-SiC and can also be reduced or eliminated by thermal annealing and other methods. A C vacancy (*V_C_*) is the most common intrinsic defect in 4H-SiC, which can act as a carrier trap, increasing the leakage current and reducing the minority carrier lifetime and device efficiency and reliability. Impurities refer to the defects caused by the incorporation of foreign atoms or molecules, such as N, aluminum (Al), vanadium (V), and boron (B), which can be introduced by doping, diffusion, and adsorption. Intentional doping, such as with N or Al, will change the carrier concentration in 4H-SiC, forming n-type or p-type semiconductors and thereby adjusting the conductivity of the material. Unintentional impurities are often related to the growth environment, such as H and O caused by an impure vacuum environment. In addition, intrinsic defects or impurities may form complexes with each other, such as C clusters (formed by interstitial C atoms gathering), *V_C_*-*V_Si_* (*VV*), *V_C_*-*C_si_* (*CAV*) [133], *N_C_*-*V_Si_* (named the *NV* center and which is famously in diamonds) [134], etc., which further affects the electrical performance of 4H-SiC devices. The existence of intrinsic defects and impurities introduces deep levels, which affects the carrier transport, recombination, and capture processes, thereby changing the conductivity, carrier lifetime, mobility, radiation resistance, and other properties. On the other hand, defects such as the divacancy, *CAV*, and *NV* exhibit degenerate electronic ground states and can be controlled by applying magnetic fields, excellent candidates for qubits. Therefore, in order to improve the 4H-SiC material quality and device performance, it is necessary to understand the physical mechanism of intrinsic defects and impurities and develop efficient methods to control and optimize their electrical behaviors.

#### 2.3.1. Intrinsic Defects

##### Carbon Vacancy

**Figure 13:**
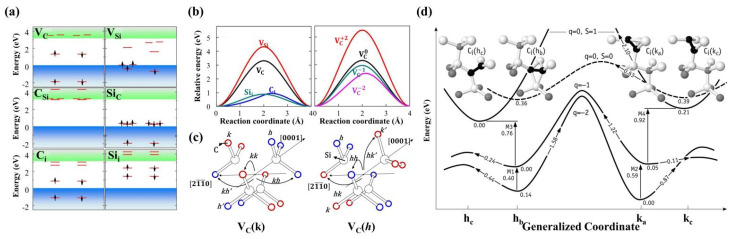
(**a**) Single-particle defect levels at *k*-sites observed in 4H-SiC, including *V_C_*, *V_Si_*, *C_Si_*, *Si_C_*, *C_i_*, and *Si_i_*. (**b**) Migration barriers of different intrinsic defects in 4H-SiC (left) and migration barriers of *V_C_* at different charge states (right). (**c**) Migration paths of *V_C_(k)* (left) and *V_C_(h)* (right) in 4H-SiC. (**d**) Atomic structures, coordinate configurations, and migration barriers of *C_i_* defects in n-type 4H-SiC at charge states q = 0, −1, and −2. (**a**,**b**) Reprinted with permission from [135], Copyright (2020) by AIP Publishing. (**c**,**d**) Reprinted from [151,152], Copyright (2019 and 2021) by American Physical Society.

When a C atom is removed from 4H-SiC to form *V_C_*, structural relaxation occurs, leading to a degraded system symmetry. The Si dangling bonds around *V_C_* then interact with each other to create defect energy levels, as depicted in Figure 13a [135]. Using the deep-level transient spectroscopy (DLTS) technique, two kinds of deep-level defects formed during the growth process of 4H-SiC epitaxial layers can be detected, namely *Z*_1/2_ and *EH*_6/7_ located at *E_C_* − 0.63 eV and *E_C_* − 1.65 eV, respectively [136,137]. The *Z*_1/2_ level is widely considered to be the key defect affecting the carrier lifetime [138,139]; thus, controlling the concentration of *Z*_1/2_ is crucial for achieving high-performance devices. Consistent concentrations and depth distributions of the *Z*_1/2_ and *EH*_6/7_ levels are observed under various processing conditions, including growth, electron irradiation, and annealing, suggesting a common origin [140,141]. Under an Si-rich growth condition and low-energy electron irradiation (below the threshold energy of Si atom displacement at 250 keV [140]), the concentrations of *Z*_1/2_ and *EH*_6/7_ levels increase [142,143]. Therefore, they are considered to correspond to the transition levels of *V_C_* with different charge states in 4H-SiC. Using the Laplace-DLTS method, the microstructure inducing the *Z*_1/2_ and *EH*_6/7_ levels can be further analyzed [144], and it has been found that they can be formed by the overlap of two single peaks, which may point to the *V_C_* at *k*- and *h*- sites, respectively. Table 5 shows the transition levels of *V_C_* in 4H-SiC reported by different groups. Hornos et al. [145] calculated the transition level (0/−2) of *V_C_(k)* at *k*-sites as 2.77 eV, which is very close to the energy level position of *Z*_1/2_. Son et al. [146] identified *Z*_1/2_ as the (0/−2) transition level of *V_C_*, *Z*_1_ (*E_C_* − 0.52 eV), and *Z*_2_ (*E_C_* − 0.45 eV) as the (0/−1) transition levels of *V_C_*(*h*) and *V_C_(k)*, respectively, and *EH*_6/7_ as the (+1/0) transition level of *V_C_* by comparing the results of DLTS and electron paramagnetic resonance (EPR). The *Z*_1/2_ center also exhibits a negative-*U* property, that is, the energy of the defect decreases when capturing the second electron [147]. This is due to the energy gain associated with electron pairing in the dangling bonds of the defect and the large lattice relaxation that overcomes the Coulomb repulsion of the two electrons. Theoretical calculations also support the negative-*U* property of *V_C_* in 4H-SiC [145,148,149]. However, the results from Hornos et al. [145] do not contain the negative-*U* property of *V_C_(h)* at *h*-sites, but the energy difference between the (0/−1) and (−1/−2) transition levels is less than 0.1 eV. In addition, theoretical calculations show that *V_C_*^2+^ is the main compensation center in p-type 4H-SiC [150]. When the Al doping concentration is between 10^16^ cm^−3^ and 10^19^ cm^−3^, the hole concentration is one order of magnitude lower than the Al concentration due to the compensation of *V_C_*^2+^. When the Al doping concentration exceeds 10^20^ cm^−3^, the hole concentration is only 10^19^ cm^−3^, affected by the compensation of *V_C_*^2+^ and the self-compensation of *Al_i_*^3+^.

In 4H-SiC, *V_C_* diffuses by exchanging positions with nearby C atoms within the lattice. As shown in the Figure 13b, the diffusion barriers of *V_C_* are found to be changed by the charge state, exhibiting high barrier heights in positive charge states and low barrier heights in negative charge states [135]. The results of Bathen et al. [151] further show that *V_C_* diffusion is anisotropic and has a lower energy barrier along the basal plane compared to the diffusion along the *c*-axis. Furthermore, the diffusion demonstrates planar selectivity, where the energy required for diffusion within the *h*-plane is lower than that within the *k*-plane. Figure 13c illustrates four distinct diffusion paths of *V_C_* in 4H-SiC, which are labeled as *kh’*, *kh*, *kk*, and *hh*, with the corresponding activation energies of 4.2 eV, 4.1 eV, 4.0 eV, and 3.7 eV, respectively. Notably, the *kh*-*hk* and *kh’*-*hk’* configurations exhibit inversion symmetry. *V_C_* has a high thermal stability in 4H-SiC and can remain stable even at annealing temperatures up to 1500 °C [140]; therefore, it is the main defect that affects the performance of 4H-SiC devices. At present, various processing methods have been developed to eliminate *V_C_*, such as the near-surface implantation of C ions [153], thermal oxidation [154,155], and annealing with a C-cap [156,157].

**Table 5:** Transition levels of *V_C_* reported by different groups in reference to *E_V_*. Left and right values in each unit correspond to the *k*- and *h*-site levels.

Ref	(+2/+1)	(+1/0)	(0/−1)	(−1/−2)	(+2/0)	(0/−2)
[145]	1.67/1.64	1.75/1.84	2.8/2.71	2.74/2.79	1.71/1.74	2.77/2.75
[158]	1.74/1.65	1.96/2.03	2.58/2.47	3.1/–	–/–	–/–
[159]	1.18/0.97	1.22/1.34	2.28/2.09	2.41/2.21	–/–	–/–
[160]	0.99/0.97	1.47/1.52	2.07/2.47	2.49/2.85	–/–	–/–
[150]	–/–	–/–	–/–	–/–	1.9/1.84	2.67/2.6
[148]	1.44/1.44	1.51/1.61	–/2.5	–/2.53	–/–	2.54/–

##### Silicon Vacancy

An Si vacancy (*V_Si_*) is another common defect in 4H-SiC that has remarkable quantum properties [161]. According to theoretical calculations, *V_Si_* can only exist in neutral, −1, −2, and −3 charge states [162], and the formation energy of neutral *V_Si_* is 7.5 eV under C-rich conditions [145], which is higher than that of *V_C_*. This implies that *V_Si_* has a low concentration under conventional growth conditions, but it could be significantly created by ion implantation or electron, proton, or heavy ion or neutron irradiation. Like *V_C_*, *V_Si_* has two different types, corresponding to the *h* and *k* lattice sites in 4H-SiC. These types can be distinguished by the two sharp peaks in the photoluminescence (PL) spectrum, namely, *V*_1_ and *V*_2_ [163]. *V_Si_* has coherence and optical controllability comparable to *NV* centers in diamonds, making it a potential qubit in 4H-SiC. The ground and excited states of negatively charged *V_Si_^−^* both exhibit an *S* = 3/2 spin quartet and 70 MHz ground-state zero field splitting, which has zero-phonon lines (ZPLs) at 861–917 nm and a long spin coherence time [164,165]. Many studies have suggested the potential applications of *V_Si_* in quantum computing, communication, and sensing [166,167,168,169]. Since *V_Si_* is immune to both the drawbacks of large electron–phonon interaction and fast spin dephasing, Nagy et al. [170] confirmed that *V_Si_* can be used in high-fidelity spin-to-photon interfaces. The results of Lukin et al. [165] show that *V_Si_* has an important role in integrated quantum and nonlinear photonics. Widmann et al. [171] showed that *V_Si_* can be used for quantum information processing. In the field of quantum sensing, *V_Si_* has exhibited its abilities in magnetic and temperature detections [172,173,174]. Additionally, it can also be used as a single photon source [175].

##### Carbon Interstitial

Before the C interstitial (*C_i_*), it is necessary to introduce the *M* center, which is a deep-level defect observed in n-type 4H-SiC after 2 MeV He ion implantation. It consists of four DLTS peaks, denoted as *M*_1_ (*E_C_* − 0.42 eV), *M*_2_ (*E_C_* − 0.63 eV), *M*_3_ (*E_C_* − 0.83 eV), and *M*_4_ (*E_C_* − 0.86 eV) [176]. The *M*_1_ and *M*_3_ (*M*_2_ and *M*_4_) can be grouped as *M*_1/3_ (*M*_2/4_), indicating different charge states of the same defect, and the *M*_1/3_ and *M*_2/4_ represent different configurations of the same defect. The *M* center has bistable characteristics, as its two configurations can be switched by controlling the thermal annealing and bias. For example, annealing at 450 K with zero bias can reduce the intensities of *M*_1_ and *M*_3_ and generate peaks of *M*_2_ and *M*_4_, while annealing at 340 K with a large reverse bias (−30 V) can restore the original DLTS spectrum. First-principle calculations show that the activation energies of *M*_1_, *M*_2_, *M*_3_, and *M*_4_ are close to the (−2/−1) and (0/−1) transition levels of different *C_i_* configurations. In 4H-SiC, *C_i_* exhibits diverse geometric shapes. Researchers have identified four distinct *C_i_* structures in neutral state and have illustrated their coordinate configuration diagrams, as depicted in Figure 13d [152]. Specifically, there exist two stable *C_i_* structures in the neutral state, denoted as ‘*hc*’ and ‘*hb*’ with *S* = 1, and two metastable structures labeled ‘*ka*’ and ‘*kc*’ with *S* = 0. The ‘*hb*’ structure becomes more stable when the *C_i_* is in a negative charge state. First-principle calculations were conducted to determine the transition levels of *C_i_*, and the results align well with the behavior of the *M* center. Table 6 shows the names of the transition levels and configurations of *C_i_* by different groups, which indicates that the *M* center is caused by *C_i_* [152,158,176,177,178]. However, the results of Karsthof et al. [179] are inconsistent with this conclusion. They used the thermal decomposition of C caps as a C source to introduce excessive C into the n-type 4H-SiC by thermal annealing and observed three peaks by DLTS, denoted as *E*_0.38_ (*E_C_* − 0.38 eV), *E*_0.59_ (*E_C_* − 0.59 eV), and *E*_0.7_ (*E_C_* − 0.7 eV). Although the activation energies of *E*_0.38_ and *E*_0.59_ are close to those of *M*_1_ and *M*_2_, their annealing behaviors are different, and they do not show the bistable characteristics of the *M* center. In addition, *EH*_1/3_ (*E_C_* − 0.4 eV) was also observed in low-energy electron and fast neutron irradiation, and Knežević et al. [180] found that *EH*_1_ was composed of a single emission line from *C_i_(h)* by Laplace-DLTS analysis. Alfieri et al. [181] also believed that *EH_1_* and *EH_3_* were related to *C_i_*, and the annealing activation energy of *EH*_1_ and *EH*_3_ that they obtained was 1.1 eV, close to the 0.95 eV diffusion barrier of *C_i_* [135].

##### Silicon Interstitial

There are multiple configurations of an Si interstitial (*Si_i_*) in 4H-SiC, but most of the stable configurations have formation energies exceeding 8 eV [182]. Since Si is less electronegative than C, *Si_i_* can only induce positive charge states in the bandgap. Moreover, the diffusion barriers of *Si_i_* are closely related to their charge states, such as the 3.7 and 2.7 eV corresponding to the +4 and +2 charge states, respectively [183]. The calculations by Coutinho et al. [183] showed that the +1 and +3 charge states are metastable, indicating that the *Si_i_* was also a negative-*U* defect. However, this negative-*U* effect was not discovered by the calculations of Kobayashi et al. [158].

##### Carbon Antisite

A C antisite (*C_Si_*) is formed by C atoms occupying the positions of Si atoms. First-principle calculations show that the formation energy of *C_Si_* is low, which means that it is easy to produce during the growth process. Kobayashi et al. [158] calculated the formation energies of neutral *C_Si_* at the *k*-site and *h*-site at the C-rich limit, which were 2.61 and 2.65 eV, respectively, consistent with the results of Torpo et al. [159]. Since neutral *C_Si_* occupies the lowest energies along the Fermi level moving at the bandgap, it is not introduced at the deep level and thus has little effect on the recombination and scattering of carriers. However, *C_Si_* often forms complexes with other defects, such as *CAV* and the antisite-interstitial pair (*C_Si_*-*C_i_*) [133,177,184]. These complexes can change the charge state of *C_Si_* and induce new trap levels at the bandgap, significantly affecting the electrical or optical performance of 4H-SiC devices.

##### Silicon Antisite

A Si antisite (*Si_C_*) in 4H-SiC also has low formation energies, such as 4.58 eV and 4.63 eV corresponding to the neutral *Si_C_*(*k*) and *Si_C_*(*h*) [158]. *Si_C_* has only one (+1/0) transition level in the bandgap, and the values of *Si_C_*(*k*) and *Si_C_*(*h*) are *E_V_* + 0.33 eV and *E_V_* + 0.34 eV, which are very similar with each other [158]. By the EPR spectra, positively charged *Si_C_^+^* could be observed experimentally in p-type 4H-SiC with a spin *S* = 1/2 [185]. In addition, the strong temperature dependence of the *g* value and hyperfine coupling constant of this SiC trap center indicates that there are considerable lattice relaxations near the defect [185].

#### 2.3.2. Impurities

##### Nitrogen

N is a widely used n-type dopant in 4H-SiC since it can enhance the electrical properties of 4H-SiC by increasing its conductivity and carrier mobility. N doping can achieve a precisely controlled electron concentration in 4H-SiC ranging from 10^14^ to 10^20^ cm^−3^, which allows for various applications of 4H-SiC in high-temperature, high-power, and high-frequency devices [186]. N doping and substituting C atoms (*N_C_*) in 4H-SiC exhibits the lowest ionization energy. The ionization energy also depends on the lattice sites, and the corresponding values for *N_C_(k)* and *N_C_(h)* are 0.092 eV and 0.052 eV [187,188,189]. *N_C_* can diffuse in 4H-SiC by exchanging with neighboring C atoms. However, the diffusion barrier for this process is notably high. Interestingly, when a C-split interstitial (two C atoms share the same lattice site) exists near *N_C_*, *N_C_* can diffuse through the replacement of one C atom, and the energy barrier of this diffusion process can be reduced to only 2.5 eV [190]. On the other hand, N doping also brings challenges and limitations for 4H-SiC devices. For example, it is found to weaken the bond strength and contribute to deformation in 4H-SiC, which facilitates the glide and piling up of basal plane dislocations in nanoindentated N-doped 4H-SiC [191]. It reduces the hardness, elastic modulus, and fracture toughness of 4H-SiC, challenging the mechanical stability and reliability [192].

##### Aluminum

Aluminum (Al) is the most commonly used dopant to create p-type 4H-SiC, which forms the shallowest acceptor levels and possesses the highest solubility (~10^21^ cm^−3^) [189]. But it still has some drawbacks and limitations compared to N doping. Al preferentially substitutes Si in 4H-SiC (*Al_Si_*). The acceptor levels (0/−1) of *Al_Si_(h)* and *Al_Si_(k)* have a very similar ionization energy of about 0.23 eV (the difference is less than 0.01 eV). This value is much larger than that of N doping, indicating a declined activation rate in Al doping. Due to the high ionization energy, Al is often incompletely ionized in 4H-SiC, and this phenomenon is further deteriorated as the Al concentration increases. For example, Wang et al. [186] observed that when the concentration of Al doping in 4H-SiC increased from 10^14^ cm^−3^ to 10^19^ cm^−3^, the ionization ratio decreased from 90% to 5% at room temperature. Even at a temperature up to 800 K, a model proposed by Darmody et al. [193] predicted an Al ionization ratio of no more than 30% at high doping concentrations. Although they observed an increase in the hole-to-Al concentration ratio at doping levels above 10^20^ cm^−3^, they attributed the increase to the variable range hopping conduction mechanism instead of the ionization ratio increase.

**Figure 14:**
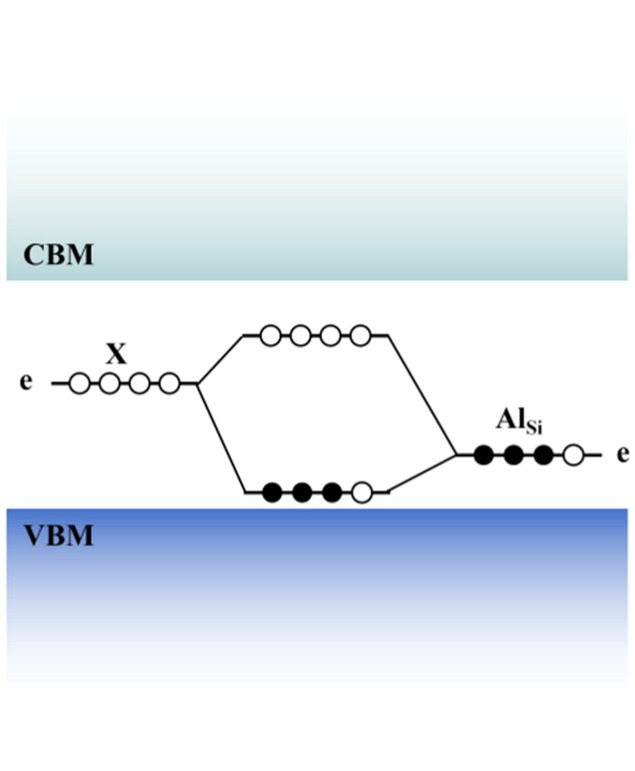
Schematic illustration of level repulsion to reduce the (0/−1) transition energy of *Al_Si_* in 4H-SiC. Reprinted from [186], Copyright (2023) by AIP Publishing.

The high ionization energy of Al in 4H-SiC is an important factor affecting the doping efficiency. One way to improve the doping efficiency is to co-dope with group-IVB elements, such as titanium (Ti) [194]. As shown in Figure 14, the double degenerated *e* states of *Al_Si_* are occupied by three electrons. When group-IVB defects with empty *e* states are combined with *Al_Si_*, a shallow acceptor level close to the VBM is obtained due to the repulsive forces between defect states of the same symmetry. With this co-doping method, it is promising to reduce the ionization energy of Al by nearly 50%. In addition, Al doping depends on the C coverage on the 4H-SiC surface, which is influenced by the growth rate, C/Si ratio, and pressure. Al doping is also higher on the Si-face than on the C-face because of the different bonding and desorption rates [195].

##### Boron

In 4H-SiC, Boron (B) impurities are often introduced unintentionally via graphite equipment during the chemical vapor deposition (CVD) process and form two types of acceptor energy levels, denoted as shallow B center (*E_V_* + 0.3 eV) and deep B center (*E_V_* + 0.65 eV), respectively [196]. A theoretical calculation shows that the two centers may correspond to the (0/−1) transition levels of substitutive B at Si and C sites (*B_Si_* and *B_C_*), which are 0.32–0.34 eV and 0.6–0.63 eV above the VBM [197]. When employing minority carrier transient spectroscopy (MCTS) and time-resolved photoluminescence, it was observed that the B-related defects critically impacted the carrier lifetime, particularly when the *Z*_1/2_ defect levels were low [198]. *B_C_* may be the main defect responsible for this phenomenon. In n-type 4H-SiC, *B_C_* is negatively charged and thus acts as hole trap to limit hole mobilities and lifetimes through capture and recombination activities.

##### Iron

Iron (Fe) is a possible candidate to obtain semi-insulating SiC. By electrical characterizations of Fe-doped n-type and p-type 4H-SiC epitaxial layers using the DLTS and MCTS techniques, it was found that Fe-related defects could affect the minority carrier lifetime and potentially influence the material’s electrical conductivity and photoelectric performance [199]. Particularly in n-type materials, Fe doping changes the capture cross-sections of electrons and holes. The study also observed the doping effects of Fe in n-type and p-type buffer layers, revealing the process of Fe-related carrier capture assisted by multi-phonon emission. Furthermore, Fe-doped 4H-SiC epitaxial layers, which are fabricated at lower temperatures using metal-organic CVD, demonstrate a notable trend: as the concentration of t-butylferrocene increases, the free carrier concentration progressively diminishes [200]. This leads to the formation of insulating SiC epitaxial layers with a characteristic free carrier concentration of 10^9^ cm^−3^ and a resistivity exceeding 10^8^ Ω·cm.

##### Nickel

Nickel (Ni) doping in 4H-SiC is characterized by a preferential substitutional occupation at the Si sites (*Ni_Si_*), which is associated with the lowest formation energy among possible doping configurations [201]. This doping process can greatly increase the dielectric loss and static dielectric constant, showing the application prospects in the field of optoelectronic and microelectric devices. Another study also pointed out that the magnetic moment of 4H-SiC could be enlarged by Ni doping. The authors attributed this enlargement to the hybridization between Ni 4*s* and C 2*p* orbitals and declared its potential application in dilute magnetic semiconductors in spintronic devices [202].

##### Vanadium and Hydrogen

High-purity semi-insulated (HPSI) 4H-SiC is also a key semiconductor material for high-frequency devices. Due to unintentional doping of impurities such as N and B, it is a common method to compensate electrons or holes by doping V. V is the amphoteric impurity in 4H-SiC and establishes acceptor and donor levels within the upper and lower half of the bandgap. Specifically, the (+4/+3) acceptor level is located at *E_C_* − 1.11 eV, and the (+5/+4) donor level is located at *E_C_* − 1.57 eV [203]. In addition, H doping is an effective strategy to suppress unintentional N doping during the physical gas phase transport growth of 4H-SiC. A study suggested that the interactions between H and N on 4H-SiC’s growth surface may yield stable ammonia (NH₃), reducing the chemical potential of N and consequently increasing its formation energy as an impurity into 4H-SiC [204].

#### 2.3.3. Complexes

##### Carbon Clusters

In SiC, *C_i_* exhibits a relative low diffusion barrier, which varies from 0.5 to 1.4 eV depending on its charge state. The barrier promotes a high degree of diffusion mobility for *C_i_* within the SiC bulk or towards its surface [205]. During this diffusion process, *C_i_* can aggregate to form various C polymers, including the dimer *C*_2_, or more extensive C clusters denoted as *C_x_*, where ‘*x*’ represents the number of C atoms polymerized.

SiC distinguishes itself from other wide bandgap semiconductors through its innate capacity to form an SiO_2_ layer via thermal oxidation [206]. This characteristic is seamlessly compatible with the established Si-based metal-oxide-semiconductor field-effect transistor (MOSFET) fabrication processes [207]. Nonetheless, it is important to note that the thermal oxidation method can potentially impair the SiC surface and subsequent device performance [208]. While the Hall mobility in bulk SiC is notably high, the *μ_FE_* in manufactured MOSFETs can experience a drastic reduction, sometimes by nearly a hundredfold, which is closely related to different oxidation processes [209,210,211]. The primary cause of performance degradation in SiC MOSFETs is the formation of interface states during thermal oxidation, and the interface states are often generated by excessive C atoms at the interface that can merge into clusters with each other [205,208,209,212,213,214,215]. Research indicates that wet oxidation surpasses dry oxidation in enhancing the device performance, likely due to two factors: the substantial reduction of the reaction barrier, facilitating interface reactions, and the H atoms generated during the process, which may promote interface quality via passivation [209,216].

**Figure 15:**
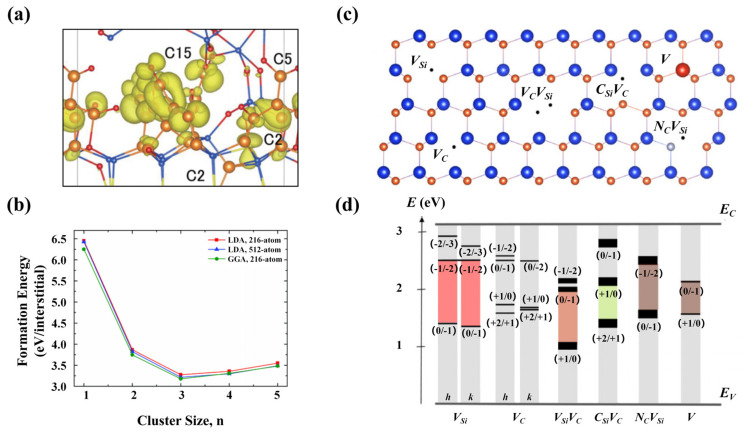
(**a**) SiO_2_/SiC interface structure with C clusters. Si, O, C, and C clusters are represented by blue, red, yellow, and orange balls. (**b**) Formation energies of neutral C clusters under different exchange-correlation functionals and unit cell volumes. (**c**) Point defects in 4H-SiC, including *V_Si_*, *V_C_*, *V_C_V_Si_* (*VV*), *C_Si_V_C_* (CAV), *N_C_V_Si_* (*NV*), and V impurity. (**d**) Transition levels of *V_Si_*, *V_C_*, *VV*, *CAV*, *NV*, and V impurity in 4H-SiC. (**a**) Reprinted from [209], Copyright (2019) by AIP Publishing. (**b**) Reprinted from [219], Copyright (2012) by American Physical Society. (**c**,**d**) Reprinted from [220], Copyright (2021) by Wiley-VCH GmbH.

The oxidation of SiC results in the formation of SiO_2_, a process that concurrently involves the reaction of C with O, leading to the release of CO or CO_2_ [209,211,217]. Nevertheless, a fraction of C atoms may not react with O. Instead, they may accumulate at the interface to form C clusters, which has been verified through first-principle MD simulations and experimental techniques like atomic force microscopy and Raman spectroscopy [210,218]. Figure 15a shows the local structure of C clusters of different sizes at the 4H-SiC interface [209]. The bonds within C clusters can be either *sp*^2^ or *sp*^3^ hybridization [209,211,217], and their dimensions decide the quality of the 4H-SiC surface [214].

The presence of point defects on 4H-SiC surfaces, such as *C_i_*, *C_Si_*, and *V_C_*, make it easier for the corresponding defect sites to form large C polymers, such as *C*_6_ clusters. Conversely, smaller clusters like *C*_2_ tend to be generated away from defects [214]. As the O concentration decreases during thermal oxidation, C atoms are inclined to merge into more complex clusters. Note that the C clusters are usually very stable at the SiC/SiO_2_ interface. The dissociation energy of *C*_2_ is estimated at 5–6 eV, suggesting its stability even under elevated temperatures. The research of Jiang et al. [219] revealed that *C*_3_ was also remarkably stable.

Following thermal oxidation, the emergence of a new defect energy level at the HK0 center was experimentally discerned in p-type 4H-SiC, with a location of *E_V_* + 0.79 eV [221]. The HK0 center necessitates annealing at temperatures exceeding 1400 °C within an Ar atmosphere. The annealing dynamics and energy level variations imply its association with the (+1/0) transition level of *C_2_*. Devynck et al.’s. [222] computations revealed the transition levels for *C_2_* at (+2/+1), (+1/0), (0/−1), and (−1/−2) located at *E_V_* + 0.49 eV, *E_V_* + 0.71 eV, *E_C_* − 0.23 eV, and *E_C_* − 0.04 eV, respectively. Wenbo Li et al. [207] also showed the transition level of *C_2_* (−1/−2) in 4H-SiC located at *E_C_* − 0.1 eV. Furthermore, it has been ascertained that the dihedral angle engendered by *C_2_* influences the positions of defect energy levels [206]. Kaneko et al. [206] reported that due to the distortion of the local structure, the defect levels of the C clusters have a wider distribution near the VBM and CBM. The energy levels formed by C clusters in the 4H-SiC bandgap will trap electrons, leading to the Fermi level pinning effect [209].

The high formation energies of C clusters are inconsistent with the substantial defect density observed in experiments [211,217]. Kobayashi et al. [158] estimated that the formation energy for neutral *C*_2_ was about 5 eV, whereas for *C*_4_, it increased to 13 eV, indicating the small probability of generating C clusters. This conclusion is supported by the results of Jiang et al. [219], as shown in Figure 15b. In the neutral state, *C*_3_ has the lowest formation energy of about 3.2 eV/atom. However, investigations by Zhang et al. [211,217] suggested that the formation energies for *C*_6_ and *C*_14_ could be reduced to 0.77 eV/atom and 0.29 eV/atom in proximity to the SiO_2_ interface or within the SiO_2_ bulk. These C clusters with reduced formation energies could be the main reason for the large amount of interface states.

##### Other Complexes

Complex defects in SiC, such as *CAV*, *NV*, and *VV* shown in Figure 15c, are regarded as promising candidates for spin qubits due to their remarkable spin properties [220]. Figure 15d illustrates the transition levels of these defects, highlighting the energy range of defect charge states suitable for quantum applications. In 4H-SiC, *CAV*, *NV*, and *VV* all exhibit four different configurations: *kk*, *kh*, *hh*, and *hk*. These distinct configurations result in subtle differences in transition energy levels and ZPL properties. For instance, the ZPL of *NV* lies within the range of 0.966–1.056 eV [134]. In the following, we take the *CAV* defect as an example to introduce its physical properties associated with quantum applications.

Experiments verified the existence of *CAV* defects after annealing irradiated samples at 600 °C [223]. Some research groups have also reported a similar phenomenon without irradiation but at 700 to 750 °C [224]. In the neutral state, the barrier to transform *V_Si_* into *CAV* was estimated to be 1.7 eV [225]. Recently, a first-principle MD simulation study revealed that within the temperature range of 1000–1500 K, C atoms near *V_Si_* exhibited a tendency to migrate to the vacancy position, forming *CAV* defects [226]. It also predicted that a backward reaction could occur at temperatures above 1500 K. Notably, Bockstedte et al. [227] had already predicted the possibility of dissociating *CAV* into the original *V_C_* and *C_Si_* configuration after crossing an energy barrier of 2.4 eV. *CAV* defects are prevalent in p-type SiC and usually serve as important carrier compensation centers in n-type or HPSI SiC [228,229]. The *hh* and *kk* configurations of *CAV* defects are characterized by *C_3v_* symmetry, whereas the *hk* and *kh* configurations display *C_1h_* symmetry [229,230].

Energy level analyses indicate that *hh* and *kk CAV* configurations possess an *a*_1_ level at approximately 1.3 eV above the *E_V_* and a double-degenerate *e* level above the *a*_1_ level [229]. In contrast, *hk* and *kh* configurations feature an *a*’ level at roughly 1.2 eV above the *E_V_* and two closely spaced *a*′ and *a*″ levels due to lower symmetry in the upper part of the bandgap. The *a*_1_ level primarily correlates with *C_Si_*, while the *e* level is associated with *V_C_* within the complex defects. The p-type 4H-SiC post-irradiation experimental results show that the *CAV* and *V_C_* concentrations are comparable and exhibit similar annealing behavior, indicating that they share a similar thermal stability and electronic levels.

Neutral *CAV* defects exhibit two spin states: *S* = 1 and *S* = 0. Szász et al. [230] derived hyperfine parameters and zero-field splitting, proposing that coherent spin manipulation in n-type 4H-SiC via optical excitation is feasible. Consequently, *CAV* emerged as a robust candidate for quantum information processing [133,230]. Furthermore, positively charged *CAV* can be intentionally designed as single-photon emitters in the red spectral region. Notably, *CAV* in SiC has been reported to emit 700 nm bright luminescence at room temperature [231]. The saturation emission rate of isolated *CAV* surpasses 2000 kc/s, outperforming other color centers in SiC. Additionally, the charge state of *CAV* can be dynamically tuned from positive to neutral using photons with wavelengths below 539 nm. With elaborate material purity design, even if *CAV* is at the positive charge state initially, effective conversion to the neutral charge state can be achieved through optical excitations.

First-principle calculations based on the HSE06 hybrid functional predict that the transition levels (+2/+1) and (+1/0) for CAV defects are located at *E_V_* + (1.28 to 1.36) eV and *E_V_* + (2.09 to 2.25) eV, respectively [230]. These theoretical predictions align well with experimental findings. For instance, photo-EPR measurements have identified the transition level (+1/0) at *E_C_* − 1.1 eV [133]. Additionally, through photosensitive current transient spectroscopy and EPR, researchers have observed the transition levels (0/−1) and (+1/0) at *E_C_* − 0.72 eV and *E_C_* − 1.1 eV, respectively [184]. Considering that the bandgap of 4H-SiC is about 3.26 eV, the experimental data agree with the theoretical calculations well.

### 2.4. Post-Processes

#### 2.4.1. Carrier Lifetime

**Figure 16:**
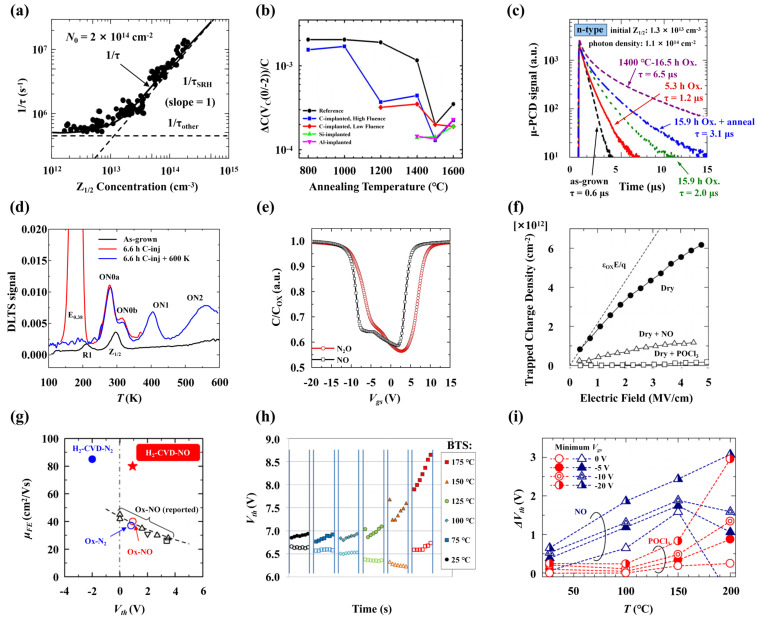
(**a**) Relationship between the inverse of carrier lifetime and *Z*_1/2_ concentration in n-type 4H-SiC epilayers. (**b**) *V_C_* (0/−2) peak amplitude variations in the C-, Al-, and Si-implanted samples plotted against the annealing temperature and obtained from DLTS measurements. (**c**) Carrier lifetimes of as-grown, oxidized, and oxidized-annealed 96-μm 4H-SiC epitaxial layers measured using differential microwave photoconductance decay (μ-PCD) at room temperature. The oxidation temperature is 1300 °C, except for the signal labeled “1400 °C.” (**d**) DLTS spectra showing the effect of C-injection after 6.6 h annealing with a C-cap. (**e**) C-V characteristics collected for 4H-SiC MOSFETs annealed in NO and N_2_O atmospheres at 1 kHz. (**f**) Density of trapped electrons as a function of the electric field across the oxide during charging. (**g**) MOSFETs’ *Vth* and *μ_FE_* improvements by the H_2_-CVD-NO process. (**h**) *Vth* variations during positive and negative stresses (±15V) measured at different temperatures (from 25 to 175 °C). (**i**) *Vth* as a function of measurement temperature and minimum *V_gs_* for POCl_3_- and NO-annealed MOSFETs. (**a**) Reprinted from [233], Copyright (2008) by Wiley-VCH GmbH. (**b**,**f**) Reprinted from [237,238], Copyright (2010 and 2015) by AIP Publishing. (**c**) Reprinted from [155], Copyright (2012) by American Institute of Physics. (**d**) Reprinted from [156], Copyright (2024) by Elsevier Ltd. (**e**) Reprinted from [239], Copyright (2021) by Elsevier B.V. (**g**) Reprinted from [240], Copyright (2021) by The Japan Society of Applied Physics. (**h**,**i**) Reprinted from [241,242], Copyright (2015) by IEEE.

In n-type 4H-SiC, extensive studies have consistently identified the *Z*_1/2_ and *EH*_6/7_ trap states as recombination centers and critical defects that significantly impact carrier lifetimes [138,139,232,233,234,235]. Figure 16a illustrates the inverse of the carrier lifetime as a function of the *Z*_1/2_ trap concentration in the n-type 4H-SiC epitaxial layer [233]. Notably, at high concentrations (>10^13^ cm^−3^), the inverse of the carrier lifetime demonstrates an approximately linear correlation with the *Z*_1/2_ trap. In this scenario, the carrier lifetime is predominantly influenced by Shockley–Read–Hall recombination due to the *Z*_1/2_ trap [236]. Conversely, at low concentrations, it is also influenced by other processes, such as surface, substrate, and Auger recombinations.

To achieve 4H-SiC power electronic devices with longer carrier lifetimes, faster response speeds, and higher reliability, it is necessary to reduce the concentration of *Z*_1/2_ and *EH*_6/7_ traps, which are believed to be closely related to the *V_C_* defect. Currently, many technological methods have been developed to reduce the *V_C_* concentration in the 4H-SiC epitaxial layer, and the core concept is annealing, such as high-temperature annealing after near-surface ion implantation [155,237], thermal oxidation [155,235,243,244,245], and with a C-cap [156,157]. The main purpose of these methods is to introduce additional C atoms to promote the annihilation of *V_C_* defects.

In the investigation of near-surface ion implantation, Kawahara et al. [155] demonstrated that the concentration of *V_C_* can be significantly reduced to below detectable limits through C ion implantation followed by annealing in an Ar environment at 1500 °C for 20 min. Note that elevating the annealing temperature beyond 1700 °C will regenerate *V_C_* defects. Ayedh et al. [237] observed an interesting result that the implantation of Al and Si ions resulted in a more pronounced decrease in the *V_C_* concentration compared to C ion implantation, as shown in Figure 16b. They believed that the species of the implanted ions played a negligible role in the *V_C_* annihilation, since the concentration of displaced C atoms exceeded the implanted ions by several orders of magnitude. Therefore, the higher efficiencies for Al and Si ions can be understood as their high energy densities to induce collision cascades and promote the annihilations between *V_C_* and *C_i_* defects.

During the thermal oxidation process, Si atoms on the surface of 4H-SiC form SiO_2_ with O atoms. Simultaneously, most C atoms escape in the form of CO. However, a fraction of C atoms remains at the interface and diffuses into the 4H-SiC bulk region, where they can annihilate *V_C_*. Figure 16c shows the improvement in carrier lifetimes in 4H-SiC epilayers by increasing the temperature and oxidation time [155]. Ichikawa et al. [244] achieved an impressive charge carrier lifetime of up to 33.2 μs in a 220 μm depth n-type 4H-SiC epitaxial layer by combining thermal oxidation at 1400 °C for 48 h with surface passivation using NO. The research of Okuda et al. [245] also showed that the carrier lifetime could be effectively improved by combining thermal oxidation with H passivation in p-type 4H-SiC.

The C-cap can be established through the spin-coating of photoresist at the front and back surfaces of 4H-SiC and graphitizing through a heat treatment at 900 °C for 10 min [156]. The subsequent annealing phase within the Ar atmosphere promotes the diffusion of C into the 4H-SiC bulk. This method is effective to eliminate *V_C_*, as shown in Figure 16d, where the Z_1/2_ and EH_6/7_ peaks disappear after treatment. In addition, medium-temperature annealing at 1500 °C in a C-rich environment is also a simple and effective method to reduce the *V_C_* concentration to 10^11^ cm^−3^ [157].

Although the annealing method can reduce the *V_C_* concentration in 4H-SiC, it is important to note that excessive C injection can also result in new defects, as illustrated in Figure 16d. Researchers have identified several novel energy levels using DLTS, including ON0a (*E_C_* − 0.58 eV), ON0b (*E_C_* − 0.68 eV), ON1 (*E_C_* − 0.89 eV), and ON2 (*E_C_* − 1.15 eV) [155,156]. Note that the Z_1/2_ and ON0a traps have similar energy level positions, but their apparent electron capture cross-sections (σ_n, app_) are quite different (5.1 × 10^−15^ cm^2^ for ON0a and 1.4 × 10^−14^ cm^2^ for Z_1/2_), indicating that they do not have the same defect origin [156]. These energy levels have been associated with C-related defects. Further investigations are necessary to assess the potential adverse impacts of these new defects on device performance.

On the other hand, a long carrier lifetime will also increase the switching loss of 4H-SiC devices. Therefore, it needs to be controlled according to the requirement of the application equipment. The carrier lifetime can be controlled by electron irradiation [139,233], annealing temperature [235], and additional carrier recombination centers [246]. Compared with electron irradiation, thermal oxidation and then Ar annealing at proper temperatures may be a better solution, since the space uniformity of *V_C_* in 4H-SiC can be obtained by controlling the annealing temperature. In contrast, electron irradiation will lead to higher and lower *V_C_* concentrations at the surface and bulk. Another effective method to control carrier lifetime is V doping in order to generate recombination centers. It has been shown that V doping can reduce the minority carrier lifetime in lightly N-doped epitaxial layer from 3 μs to 40 ns, and this value is 20 ns in the highly N-doped case, which is accompanied by a high thermal stability (>1700 °C).

#### 2.4.2. Mobility

Theoretically, 4H-SiC bulk material has high electron mobilities [247]. However, during the fabrication of MOSFETs, a notable decrease in *μ_FE_* is observed. This reduction is primarily attributed to the high interface state density (*D_it_*) present at the SiC/dielectric interface. These states serve as trap centers for both electrons and holes, accelerating the charge carrier scattering and consequently reducing the mobility. The C clusters formed at the SiC/SiO_2_ interface during the thermal oxidation process are believed to lead to significantly elevated *D_it_*, posing a challenge to device performance [239]. In commercial applications, carrier mobilities in 4H-SiC MOSFETs can be predominantly enhanced via nitridation processes, such as treatments by N_2_O [239,248], NO [238,239,249,250], and N_2_ [251,252]. N often eliminates interface states by forming Si-N_3_ or Si-N_4_ [253,254]. Under similar experimental conditions, a 55 cm^2^/V·s device mobility can be obtained in a NO atmosphere, while it is only 20 cm^2^/V·s in a N_2_O atmosphere, indicating that NO is a better solution [239]. From the capacitance–voltage (CV) measurement in Figure 16e, a lower *D_it_* is detected after NO annealing due to the steeper slope of the curve. This conclusion is supported by DFT calculations, which predict more favorable reaction kinetics for NO with a low energy barrier of 0.84 eV, compared with 3.4 eV for N_2_O at the C-face of 4H-SiC [253,255]. However, the application of NO is limited due to its inherent toxicity. A study conducted by Chanthaphan et al. [252] demonstrated that for thin SiO_2_ layers (<15 nm), N_2_ annealing at temperatures above 1350 °C could show better performance than NO annealing. However, N_2_ annealing also leads to a negative *Vth* shift of 4H-SiC MOSFETs [240]. Furthermore, Wang et al. [256] introduced an innovative approach utilizing supercritical N_2_O fluid at only 120 °C to effectively reduce *D_it_* to 2.8 × 10^11^ cm^−2^eV^−1^.

In addition, nitridation can lead to additional defects, which subsequently impact the performance of 4H-SiC MOSFETs [257,258]. Yoshioka et al. [247] observed a fast interfacial state in 4H-SiC following nitridation. Similarly, Fujimoto et al. [258] demonstrated that in NO passivated devices, additional defects that became active upon exposure to UV irradiation were introduced, thereby affecting overall device behaviors. Furthermore, a finding by Kobayashi et al. [259] indicated that NO nitridation may not completely eliminate C-related defects. Instead, a highly N-doped thin slice was formed on the epitaxial layer surface. Due to the band bending and the underestimation of energy differences between the surface Fermi level and *E_C_*, the defect densities are apparently lower.

Other annealing methods beyond nitridation also have been widely considered. Compared with nitridation, Ar annealing at 1500 °C demonstrates similar performance, but without inducing fast interfacial states [260]. Additionally, phosphoryl chloride (POCl_3_) annealing is known to enhance interface quality and reduce *D_it_*. In a study by Okamoto et al. [238], POCl_3_ annealing achieved an impressive *μ_FE_* of 89 cm^2^/V·s, as shown in Figure 16f. However, it is important to note that POCl_3_ annealing can result in the formation of P-related oxide defects, potentially impacting device stability [242].

Moreover, eschewing conventional thermal oxidation methods seems to be another effective strategy for enhancing the mobility of 4H-SiC devices. A three-step process involving H_2_ etching, CVD grown in SiO_2_, and nitridation, as demonstrated by Tachiki et al. [240], resulted in a reduction of *D_it_* to 4–6 × 10^10^ cm^−2^eV^−1^ and an increase in *μ_FE_* to 80–85 cm^2^/V·s, as illustrated in Figure 16g. Furthermore, employing a similar three-step approach of surface plasma nitridation, SiO_2_ sputtering deposition, and annealing in a CO_2_ environment yielded a small *D_it_* value of 10^11^ cm^−2^eV^−1^ [261]. In this method, the interface performance was immune to excimer UV light irradiation, demonstrating enhanced stability.

#### 2.4.3. Instability

In 4H-SiC MOSFETs, the *Vth* instability can manifest under bias voltage conditions and be significantly influenced by temperatures. This phenomenon is categorized into two distinct types: negative bias temperature instability (NBTI) and positive bias temperature instability (PBTI). Both NBTI and PBTI in 4H-SiC MOSFETs demonstrate a rapid recovery characteristic, typically within milliseconds [262]. Notably, NBTI exhibits the least temperature sensitivity, whereas PBTI shows pronounced temperature dependence, as shown in Figure 16h. Furthermore, Lelis et al. [241] indicated a positive *Vth* shift in NBTI at temperatures exceeding 175 °C. This shift is likely correlated with the generation of additional interface traps at elevated temperatures.

Recent studies have proposed various mechanisms to elucidate the instability phenomena observed in 4H-SiC MOSFETs. Defects such as *C_i_* [263], *V_O_* [241,264], and N-related defects within the SiO_2_ layer are among the primary suspects [265]. Research by Ettisserry et al. [263] indicated that a *C_i_* defect in the SiO_2_ layer significantly contributed to instability, which could be restrained by NO annealing, leading to the formation of NCO complexes. In a neutral state, the *V_O_* defect is known to form Si-Si bonds, and the bonds can be broken by capturing holes [241]. It has been observed that the hole trap density correlates linearly with N concentration, possibly due to the formation of SiO_x_N_y_ transition layers [265].

To deal with instability issues, several strategies have been suggested. For instance, pre-doping 4H-SiC with O allows for a reduced *Vth* shift [266]. Additionally, POCl_3_ and NO annealing have emerged as effective alternatives [242,263,267]. Devices subjected to POCl_3_ annealing exhibit a smaller *Vth* shift than NO annealing under NBTI and PBTI conditions at temperatures below 200 °C, as shown in Figure 16i [242]. However, it is noteworthy that at temperatures reaching 200 °C, a significant *Vth* shift is observed in devices treated with POCl_3_. The underlying causes of instability in NO and POCl_3_ annealed devices are different and need further investigation.

### 2.5. Application

Over the past few decades of technological advancement, Si-based power devices have become an indispensable part of power electronic systems, bringing obvious economic benefits to various industries. However, as technology continues to advance, the performance potential of Si-based devices is reaching its limit, especially in high-voltage applications. For example, in 2023, the rated voltage of commercial Si-based MOSFETs on the market typically did not exceed 950 V, which is clearly insufficient to meet the demands of emerging applications such as 800 V electric vehicles and 1500 V solar inverters [268].

**Figure 17:**
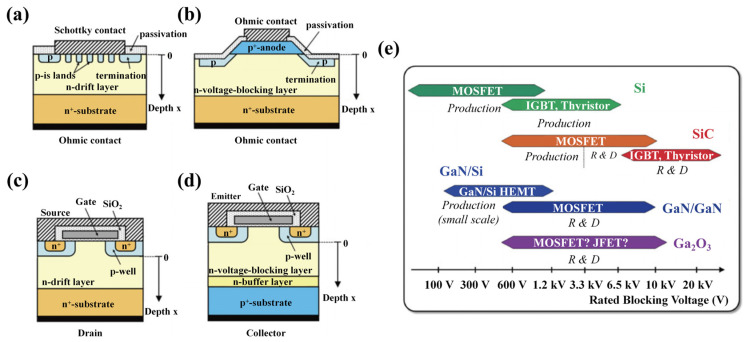
Schematic structures of typical SiC power devices, such as the (**a**) Junction-Barrier Schottky diode (modified structure of a Schottky barrier diode), (**b**) PiN diode, (**c**) planar-type vertical MOSFET, and (**d**) insulated gate bipolar transistor (IGBT). (**e**) Major application ranges in terms of the device blocking voltage for Si, SiC, GaN, and Ga_2_O_3_ power switching devices. (**a**–**e**) Reprinted from [276], Copyright (2022) by The Japan Academy.

In the quest for solutions with higher voltages and better performance, SiC devices have emerged as a powerful candidate. The unique physical properties of SiC, such as a high breakdown electric field and excellent thermal stability, enable it to achieve the abilities of blocking high voltages and reducing switching and conduction losses. Figure 17e compares the blocking voltages of Si, SiC, GaN, and Ga_2_O_3_ power devices, highlighting the advantages of SiC devices over Si devices. These characteristics perfectly meet the demands of a higher efficiency, greater power density, and faster switching speeds in power electronic devices [236].

Since Infineon launched the first commercial SiC Schottky diode to the market in 2001, the development of SiC devices has made rapid progress [269]. Today, devices made of SiC, such as diodes, MOSFETs, junction field-effect transistors (JFETs), and bipolar junction transistors (BJTs), have been widely applied in key circuit components such as inverters, transformer rectifier units, and DC-DC converters, becoming the core technology in multiple fields including solar energy, electric vehicles, aerospace, and railway traction [270,271,272,273,274,275]. The typical structures of common SiC power devices are shown in Figure 17a–d [276]. Currently, SiC devices ranging from 600 V to 3300 V are available on the market, and devices with a voltage rating of 10 kV or even higher are being actively developed, indicating a bright future for SiC technology [268].

Currently, major companies are working on popularizing SiC devices to the electric vehicle area. With the development of a battery pack voltage from 400 V to over 800 V, SiC devices have garnered significant attention [277]. They exhibit wide-ranging applications in drive inverters, on-board chargers (OBCs), and direct current charging station modules. Simulation results indicate that using a full SiC inverter can reduce energy consumption in electric vehicles by approximately 5% and increase the endurance by 5%. The typical structure of an OBC consists of a front-end power factor correction (PFC) circuit and a rear-end DC-DC output circuit [278]. Research has demonstrated that a SiC-based 5 kW three-phase full-bridge PFC achieved an efficiency rate of 98.8% [279], and a SiC-based 3.6 kW totem-pole PFC achieved 99.3% efficiency [280]. Additionally, a 3.5 kW DC-DC converter using SiC operated at a full-load efficiency of 95.5% at 700 V [278]. A proposed architecture by Saha et al. [281] utilized SiC MOSFETs in a medium-voltage grid-connected transformer for rapid charging stations. This architecture can fully charge a large-sized electric vehicle in 49.5 min, two medium-sized electric vehicles in 28 min, or four small-sized electric vehicles in 16 min.

All-electric aircrafts are an essential choice for advancing sustainable development in the aviation industry [274,275]. As the global aviation sector continues to expand, its environmental impact grows more pronounced. The all-electric aircraft offers the potential to reduce C emissions and improve energy efficiency. However, electric aircrafts require megawatt-level power, which needs significant advancements in power electronics integration, performance, efficiency, component size, and weight reduction. To realize these requirements, SiC devices may be a choice. In 2022, the first all-electric aircraft named e-Sling successfully completed its inaugural flight in Switzerland, covering approximately 180 km. Notably, it employed a SiC modular battery system with an impressive energy density of 196 Wh/kg.

SiC also has many applications in the field of detectors or sensors. Li et al. [282] successfully fabricated solar-blind photodetectors utilizing a top-down methodology via anodic oxidation of 4H-SiC single crystals. The outcomes indicated a superior solar-blind detection performance characterized by enhanced sensitivity to ultraviolet radiation at wavelengths of 275 and 365 nm, coupled with a notable response time swift of 47.3 ms. Mo et al. [283] proposed a highly linear temperature sensor based on 4H-SiC p-n diodes. The diode has an excellent linear response to temperature from room temperature to 600 °C at a constant current bias.

In the traction inverter systems of commuter trains, the integration of 3.3 kV full SiC MOSFET power modules enhances the operational efficiency and yields substantial electrical energy savings. Empirical studies suggest that SiC modules can diminish electrical energy losses by up to 59%. Additionally, the elevated switching frequency and superior thermal endurance of SiC power modules can simplify the system packaging, promoting a more compact and eco-friendly traction drive system [284].

SiC also emerges as an exceptionally well-suited material to meet the development direction of photovoltaic inverters towards greater power, higher efficiency, and smaller size. Jinkui He et al. [285] presented a variable-resistance gate driver design using SiC MOSFET-based 1500 V photovoltaic inverters, enhancing the reliability and lifetime performance of high-voltage photovoltaic applications.

Furthermore, Chen et al. [286] proposed the architecture of a 150 kW/1500 V SiC-based grid-forming photovoltaic synchronous generator (PVSG). This innovative PVSG combined a 150 kW three-phase photovoltaic inverter, a 100 kW DC-DC converter, and a 2.4 F supercapacitor module to provide energy storage and inertia support. The design achieved an inverter peak efficiency of 99.1% and a DC-DC converter efficiency of 99.5%, showing exceptional performance potential.

SiC technology offers abundant benefits, but its popularization still faces challenges. The successful integration of SiC devices into electrical systems needs a comprehensive understanding of the system architecture, particularly considering electromagnetic interference and thermal management concerns. Additionally, the elevated expense associated with SiC components is also an important problem hindering their popularization.

## 3. Conclusions

Wide bandgap semiconductors possess fascinating physical properties beyond Si, including the high breakdown voltage, high radiation tolerance, high thermal conductivity, fast switching speed, and reduced leakage current, making them one of the best candidates for high-power electronics to optoelectronic devices. As two representatives of amorphous and crystal wide bandgap semiconductors, the mechanical, physical, and electrical performance of a-IGZO and 4H-SiC are discussed in this review from the perspective of defects. Here, we make a short comparison between them.

a-IGZO is an amorphous material and lacks a long-range ordered crystal structure, making it naturally possess small mobilities. Although the spatially spread In 5*s* orbitals with large isotropic spherical extensions can result in large overlaps of *s* orbitals between adjacent metal atoms, the mobilities of a-IGZO are still much smaller than those of 4H-SiC. On the other hand, the crystal structure of 4H-SiC significantly enhances the difficulty of its grown technology, since temperatures exceeding 1000 °C are frequently required at annealing stages. This also hinders its compatibility with other material technologies.

Because of the amorphous property of a-IGZO and high-temperature manufacturing process of 4H-SiC, defect problems persistently impede their device applications. Defects in a-IGZO are often induced by the unbalanced local metal-to-O ratios, generating M-M and O-O bonds. The most obvious characteristic is the stability of these defects. They can be formed and eliminated at specific conditions related to the device’s operation environment, thus leading to instability issues in a-IGZO device performance under PBS and NBS conditions. In comparison, the performance deviations in 4H-SiC devices usually occur at stricter bias stress conditions and recover more rapidly. However, the performance degradation induced by defects is persistent and serious considering the high-temperature and voltage application environment. They can reduce the mobility by more than one order of magnitude or even cause breakdown of the device.

In summary, a-IGZO is favored for its use in display technologies and compatibility with flexible substrates, while 4H-SiC is often chosen for high-power and temperature applications, where its exceptional thermal and electronic properties are essential. The choice between these materials largely depends on the defect-related physical and mechanical characteristics.

## Figures and Tables

**Table 1:** Structure properties of a-IGZO.

	Ref	Bond Length (Å)			Coordination Number			Density (g/cm^3^)
		In-O	Ga-O	Zn-O	In	Ga	Zn	
Calculation	[13]	2.15	1.79	2	5.36	4.25	4.22	5.58
	[14]	2.2	2	2	5	5	4	5.71
	[15]	2.12	1.91	1.92	4.8	5	4	6.1
	[16]	–	–	–	5.24	4.9	4.41	5.77
	[17]	2.14	1.9	2	5.26	4.83	4.25	5.91
Experiment	[13]	2.11	2	1.95	4.5	4.3	4.6	5.9
	[18]	2.16	1.87	1.97	4.9	5.0	4.5	–

**Table 2:** The performance of a-IGZO TFTs using different preparation methods and metal atomic composition ratios. *μ_sat_* and *μ_FE_* represent saturation and field-effect mobilities.

Ref	Method	*W/L* (μm/μm)	Dielectric	In:Ga:Zn	Mobility (cm^2^/V·s)	*SS* (V/dec)	*I_on_*/*I_off_*	*Vth* (V)
[23]	Sputtering	(100–300)/(10–50)	SiO_2_	37:13:50	12 (*μ_sat_*)	–	10^8^	3
[27]	Sputtering	25/25	SiO_2_	2:1:2	52 (*μ_FE_*)	0.25	10^8^	1
				4:1:2	74 (*μ_FE_*)	0.29	10^8^	0.2
[28]	Sputtering	25/25	SiO_2_	3:6:2	52 (*μ_FE_*)	0.25	–	1.9
[29]	Solution	160/20	Al_2_O_3_	3:1:1	3 (*μ_sat_*)	0.073	10^6^	–
[30]	Solution	1500/100	Al_2_O_3_	5:1:1	9	0.22	10^6^	0.2
[31]	Solution	300/30	HfO_2_	9:1:2	86 (*μ_FE_*)	0.14	–	−0.3
[5]	PEALD	40/20	SiO_2_	23:14:8	74 (*μ_sat_*)	0.26	10^9^	−1.3
[32]	PEALD	40/20	SiO_2_	5:3:1	24 (*μ_sat_*)	0.29	10^8^	0.5
				64:15:22	44 (*μ_sat_*)	0.25	10^9^	−1.1

**Table 3:** Trap level positions from VBM induced by M-M bonds, bandgaps, formation energies, and atom numbers of the a-IGZO models in theoretical works.

Ref	Trap Level Position (eV)	Bandgap (eV)	Formation Energy (eV)	Atom Number
[15]	0.28–0.73	1.4	3.9–5	84
[13]	1.64	3.1	3–5.8	84
[16]	1.36–2.45	2.7	3.4–5	84
[42]	1	2.5	–	112
[39]	1.14	2	–	336
	1.51	3.1	–	168
[36]	0.89–2	2.5	–	105
[38]	0.75–1.55	2.9	0.7–5.2	490
[37]	0.8–1.2	2.9	–	112

**Table 4:** Performance of 2T0C DRAM units using IGZO by different research groups.

Ref.	Structure	*W/L* (nm/nm)	*I_on_* (μA/μm)	*I_off_* (A/μm)	Retention (s)	*SS* (mV/dec)	*Vth* (V)
[107]	Single gate	70/45	0.2	3 × 10^−19^	>400	–	−0.4
[108]	CAA	–	30	2 × 10^−7^	300	230	−3.5
[109]	Single gate	180/50	4	<10^−19^	>1000	–	0
[110]	CAA	–	33	4 × 10^−19^	–	92	−0.2
[111]	Double gate	220/14	1500	–	>300	77	–
[112]	Single gate	–	24	6 × 10^−20^	>10,000	–	1.2

**Table 6:** Transition levels of *C_i_* reported by different groups in reference to *E_V_*. Left and right values in each unit correspond to the *k*- and *h*-site levels.

Ref	(+2/+1)	(+1/0)	(0/−1)	(−1/−2)
[177]	0.3/0.17	1.62/1.48	2.22/2.53	2.49/2.8
[158]	1/0.8	1.67/1.58	2.5/2.87	–/–
[176]	0.9/0.69	1.53/1.43	2.38/2.54	2.71/2.9

## Data Availability

Data are contained within the article.

## References

[1] Hosono H., Yasukawa M., Kawazoe H. (1996). Novel oxide amorphous semiconductors: Transparent conducting amorphous oxides. J. Non-Cryst. Solids.

[2] Hosono H., Kikuchi N., Ueda N., Kawazoe H. (1996). Working hypothesis to explore novel wide band gap electrically conducting amorphous oxides and examples. J. Non-Cryst. Solids.

[3] Nomura K., Ohta H., Takagi A., Kamiya T., Hirano M., Hosono H. (2004). Room-temperature fabrication of transparent flexible thin-film transistors using amorphous oxide semiconductors. Nature.

[4] Kamiya T., Nomura K., Hosono H. (2010). Present status of amorphous In–Ga–Zn–O thin-film transistors. Sci. Technol. Adv. Mater..

[5] Sheng J., Hong T., Lee H.-M., Kim K., Sasase M., Kim J., Hosono H., Park J.-S. (2019). Amorphous IGZO TFT with High Mobility of ∼70 cm^2^/(V s) via Vertical Dimension Control Using PEALD. ACS Appl. Mater. Interfaces.

[6] Ide K., Nomura K., Hosono H., Kamiya T. (2019). Electronic Defects in Amorphous Oxide Semiconductors: A Review. Phys. Status Solidi A.

[7] Billah M.M., Hasan M.M., Jang J. (2017). Effect of Tensile and Compressive Bending Stress on Electrical Performance of Flexible a-IGZO TFTs. IEEE Electron Device Lett..

[8] Lee S., Jeon S., Chaji R., Nathan A. (2015). Transparent Semiconducting Oxide Technology for Touch Free Interactive Flexible Displays. Proc. IEEE.

[9] Won D., Bang J., Choi S.H., Pyun K.R., Jeong S., Lee Y., Ko S.H. (2023). Transparent Electronics for Wearable Electronics Application. Chem. Rev..

[10] Kase N., Kimizuka N., Miyakawa N. (2022). Recent progress of the single crystal growth of homologous (InGaO_3_)*_m_*(ZnO)*_n_*. CrystEngComm.

[11] Assenmacher W., Schnakenburg G., Michiue Y., Kanke Y., Kimizuka N., Mader W. (2014). Synthesis and crystal structure characterization of InGaZnO_4_ with a new defect structure. J. Solid State Chem..

[12] Medvedeva J.E., Bhattarai B., Buchholz D.B. (2022). Electronic Structure and Structural Randomness. Amorphous Oxide Semiconductors.

[13] Noh H.-K., Chang K.J., Ryu B., Lee W.-J. (2011). Electronic structure of oxygen-vacancy defects in amorphous In-Ga-Zn-O semiconductors. Phys. Rev. B.

[14] Nomura K., Kamiya T., Ohta H., Uruga T., Hirano M., Hosono H. (2007). Local coordination structure and electronic structure of the large electron mobility amorphous oxide semiconductor In-Ga-Zn-O: Experiment and ab initio calculations. Phys. Rev. B.

[15] Kamiya T., Nomura K., Hosono H. (2010). Subgap states, doping and defect formation energies in amorphous oxide semiconductor a-InGaZnO_4_ studied by density functional theory. Phys. Status Solidi A.

[16] Körner W., Urban D.F., Elsässer C. (2013). Origin of subgap states in amorphous In-Ga-Zn-O. J. Appl. Phys..

[17] De Meux A.D.J., Pourtois G., Genoe J., Heremans P. (2015). Comparison of the electronic structure of amorphous versus crystalline indium gallium zinc oxide semiconductor: Structure, tail states and strain effects. J. Phys. D Appl. Phys..

[18] Jia J., Suko A., Shigesato Y., Okajima T., Inoue K., Hosomi H. (2018). Evolution of Defect Structures and Deep Subgap States During Annealing of Amorphous In-Ga-Zn Oxide for Thin-Film Transistors. Phys. Rev. Appl..

[19] Hosono H. (2006). Ionic amorphous oxide semiconductors: Material design, carrier transport, and device application. J. Non-Cryst. Solids.

[20] Hosono H., Yamashita Y., Ueda N., Kawazoe H., Shimidzu K. (1996). New amorphous semiconductor: 2CdO⋅PbOx. Appl. Phys. Lett..

[21] Yasukawa M., Hosono H., Ueda N., Kawazoe H. (1995). Novel Transparent and Electroconductive Amorphous Semiconductor: Amorphous AgSbO_3_ Film. Jpn. J. Appl. Phys..

[22] Nomura K., Ohta H., Ueda K., Kamiya T., Hirano M., Hosono H. (2003). Thin-Film Transistor Fabricated in Single-Crystalline Transparent Oxide Semiconductor. Science.

[23] Iwasaki T., Itagaki N., Den T., Kumomi H., Nomura K., Kamiya T., Hosono H. (2007). Combinatorial approach to thin-film transistors using multicomponent semiconductor channels: An application to amorphous oxide semiconductors in In–Ga–Zn–O system. Appl. Phys. Lett..

[24] Kim J., Park J., Yoon G., Khushabu A., Kim J.-S., Pae S., Cho E.-C., Yi J. (2020). Effect of IGZO thin films fabricated by Pulsed-DC and RF sputtering on TFT characteristics. Mater. Sci. Semicond. Process..

[25] Kamiya T., Nomura K., Hosono H. (2009). Origins of High Mobility and Low Operation Voltage of Amorphous Oxide TFTs: Electronic Structure, Electron Transport, Defects and Doping. J. Disp. Technol..

[26] Vogt K.T., Malmberg C.E., Buchanan J.C., Mattson G.W., Brandt G.M., Fast D.B., Cheong P.H.-Y., Wager J.F., Graham M.W. (2020). Ultrabroadband density of states of amorphous In-Ga-Zn-O. Phys. Rev. Res..

[27] Barquinha P., Pereira L., Gonçalves G., Martins R., Fortunato E. (2009). Toward High-Performance Amorphous GIZO TFTs. J. Electrochem. Soc..

[28] Olziersky A., Barquinha P., Vilà A., Magaña C., Fortunato E., Morante J.R., Martins R. (2011). Role of Ga_2_O_3_–In_2_O_3_–ZnO channel composition on the electrical performance of thin-film transistors. Mater. Chem. Phys..

[29] Moreira M., Carlos E., Dias C., Deuermeier J., Pereira M., Barquinha P., Branquinho R., Martins R., Fortunato E. (2019). Tailoring IGZO Composition for Enhanced Fully Solution-Based Thin Film Transistors. Nanomaterials.

[30] Xu W., Hu L., Zhao C., Zhang L., Zhu D., Cao P., Liu W., Han S., Liu X., Jia F. (2018). Low temperature solution-processed IGZO thin-film transistors. Appl. Surf. Sci..

[31] Liu Y., Yu Y., Li T., Hu Y., Unnithan R., Skafidas E. (2023). High Performance and High Yield Solution Processed IGZO Thin Film Transistors Fabricated with Low-Temperature Annealed Hafnium Dioxide Gate Dielectric. Adv. Electron. Mater..

[32] Hong T., Kim Y., Choi S., Lim J.H., Park J. (2023). Exploration of Chemical Composition of In–Ga–Zn–O System via PEALD Technique for Optimal Physical and Electrical Properties. Adv. Electron. Mater..

[33] Nomura K., Kamiya T., Yanagi H., Ikenaga E., Yang K., Kobayashi K., Hirano M., Hosono H. (2008). Subgap states in transparent amorphous oxide semiconductor, In–Ga–Zn–O, observed by bulk sensitive x-ray photoelectron spectroscopy. Appl. Phys. Lett..

[34] Zhou Y., Wang D., Li Y., Jing L., Li S., Chen X., Zhang B., Shuai W., Tao R., Lu X. (2022). Critical Effect of Oxygen Pressure in Pulsed Laser Deposition for Room Temperature and High Performance Amorphous In-Ga-Zn-O Thin Film Transistors. Nanomaterials.

[35] Zhang W., Fan Z., Shen A., Dong C. (2021). Atmosphere Effect in Post-Annealing Treatments for Amorphous InGaZnO Thin-Film Transistors with SiOx Passivation Layers. Micromachines.

[36] De Meux A.D.J., Bhoolokam A., Pourtois G., Genoe J., Heremans P. (2017). Oxygen vacancies effects in a-IGZO: Formation mechanisms, hysteresis, and negative bias stress effects. Phys. Status Solidi A.

[37] Song H., Kang G., Kang Y., Han S. (2019). The Nature of the Oxygen Vacancy in Amorphous Oxide Semiconductors: Shallow Versus Deep. Phys. Status Solidi B.

[38] De Meux A.D.J., Pourtois G., Genoe J., Heremans P. (2018). Defects in Amorphous Semiconductors: The Case of Amorphous Indium Gallium Zinc Oxide. Phys. Rev. Appl..

[39] Han W.H., Chang K.J. (2016). Subgap States near the Conduction-Band Edge Due to Undercoordinated Cations in Amorphous In-Ga-Zn-O and Zn-Sn-O Semiconductors. Phys. Rev. Appl..

[40] Kamiya T., Nomura K., Hirano M., Hosono H. (2008). Electronic structure of oxygen deficient amorphous oxide semiconductor a-InGaZnO_4–*x*_: Optical analyses and first-principle calculations. Phys. Status Solidi C.

[41] Ryu B., Noh H.-K., Choi E.-A., Chang K.J. (2010). O-vacancy as the origin of negative bias illumination stress instability in amorphous In–Ga–Zn–O thin film transistors. Appl. Phys. Lett..

[42] Nahm H.-H., Kim Y.-S. (2014). Undercoordinated indium as an intrinsic electron-trap center in amorphous InGaZnO_4_. NPG Asia Mater..

[43] Nakashima M., Oota M., Ishihara N., Nonaka Y., Hirohashi T., Takahashi M., Yamazaki S., Obonai T., Hosaka Y., Koezuka J. (2014). Origin of major donor states in In–Ga–Zn oxide. J. Appl. Phys..

[44] De Meux A.D.J., Pourtois G., Genoe J., Heremans P. (2018). Effects of hole self-trapping by polarons on transport and negative bias illumination stress in amorphous-IGZO. J. Appl. Phys..

[45] Kamiya T., Nomura K., Hosono H. (2009). Electronic structure of the amorphous oxide semiconductor a-InGaZnO_4-*x*_: Tauc-Lorentz optical model and origins of subgap states. Phys. Status Solidi A.

[46] Kim G. (2022). Evaluation of oxygen-vacancy concentration through simulated hydrogen diffusion in amorphous In-Ga-Zn-O. Comput. Mater. Sci..

[47] Yao J., Xu N., Deng S., Chen J., She J., Shieh H.P.D., Liu P.T., Huang Y.P. (2011). Electrical and Photosensitive Characteristics of a-IGZO TFTs Related to Oxygen Vacancy. IEEE Trans. Electron Devices.

[48] Kim C.H., Jang Y.H., Hwang H.J., Song C.H., Yang Y.S., Cho J.H. (2010). Bistable resistance memory switching effect in amorphous InGaZnO thin films. Appl. Phys. Lett..

[49] Han W.H., Oh Y.J., Chang K.J., Park J.-S. (2015). Electronic Structure of Oxygen Interstitial Defects in Amorphous In-Ga-Zn-O Semiconductors and Implications for Device Behavior. Phys. Rev. Appl..

[50] Nahm H., Kim Y., Kim D.H. (2012). Instability of amorphous oxide semiconductors via carrier-mediated structural transition between disorder and peroxide state. Phys. Status Solidi B.

[51] Zhou X., Shao Y., Zhang L., Lu H., He H., Han D., Wang Y., Zhang S. (2017). Oxygen Interstitial Creation in a-IGZO Thin-Film Transistors Under Positive Gate-Bias Stress. IEEE Electron Device Lett..

[52] Sallis S., Butler K.T., Quackenbush N.F., Williams D.S., Junda M., Fischer D.A., Woicik J.C., Podraza N.J., White B.E., Walsh A. (2014). Origin of deep subgap states in amorphous indium gallium zinc oxide: Chemically disordered coordination of oxygen. Appl. Phys. Lett..

[53] Ide K., Kikuchi Y., Nomura K., Kimura M., Kamiya T., Hosono H. (2011). Effects of excess oxygen on operation characteristics of amorphous In-Ga-Zn-O thin-film transistors. Appl. Phys. Lett..

[54] Choi S., Park J., Hwang S., Kim C., Kim Y., Oh S., Baeck J.H., Bae J.U., Noh J., Lee S. (2022). Excessive Oxygen Peroxide Model-Based Analysis of Positive-Bias-Stress and Negative-Bias-Illumination-Stress Instabilities in Self-Aligned Top-Gate Coplanar In–Ga–Zn–O Thin-Film Transistors. Adv. Electron. Mater..

[55] Jeong H.-S., Cha H.-S., Hwang S.-H., Lee D.-H., Song S.-H., Kwon H.-I. (2021). Effects of Oxygen Content on Operational Characteristics and Stability of High-Mobility IGTO Thin-Film Transistors during Channel Layer Deposition. Coatings.

[56] Bang J., Matsuishi S., Hosono H. (2017). Hydrogen anion and subgap states in amorphous In–Ga–Zn–O thin films for TFT applications. Appl. Phys. Lett..

[57] Tang H., Ishikawa K., Ide K., Hiramatsu H., Ueda S., Ohashi N., Kumomi H., Hosono H., Kamiya T. (2015). Effects of residual hydrogen in sputtering atmosphere on structures and properties of amorphous In-Ga-Zn-O thin films. J. Appl. Phys..

[58] Sato A., Abe K., Hayashi R., Kumomi H., Nomura K., Kamiya T., Hirano M., Hosono H. (2009). Amorphous In–Ga–Zn–O coplanar homojunction thin-film transistor. Appl. Phys. Lett..

[59] Orui T., Herms J., Hanyu Y., Ueda S., Watanabe K., Sakaguchi I., Ohashi N., Hiramatsu H., Kumomi H., Hosono H. (2015). Charge Compensation by Excess Oxygen in Amorphous In–Ga–Zn–O Films Deposited by Pulsed Laser Deposition. J. Disp. Technol..

[60] Mattson G.W., Vogt K.T., Wager J.F., Graham M.W. (2022). Hydrogen incorporation into amorphous indium gallium zinc oxide thin-film transistors. J. Appl. Phys..

[61] Tang H., Kishida Y., Ide K., Toda Y., Hiramatsu H., Matsuishi S., Ueda S., Ohashi N., Kumomi H., Hosono H. (2017). Multiple Roles of Hydrogen Treatments in Amorphous In–Ga–Zn–O Films. ECS J. Solid State Sci. Technol..

[62] Velichko R., Magari Y., Furuta M. (2022). Defect Passivation and Carrier Reduction Mechanisms in Hydrogen-Doped In-Ga-Zn-O (IGZO:H) Films upon Low-Temperature Annealing for Flexible Device Applications. Materials.

[63] Liu G., Kong Q., Zhang D., Wang X., Zhou Z., Jiao L., Han K., Kang Y., Nguyen B.-Y., Ni K. (2024). Hydrogen-Related Instability of IGZO Field-Effect Transistors. IEEE Trans. Electron Devices.

[64] Nomura K., Kamiya T., Hosono H. (2013). Effects of Diffusion of Hydrogen and Oxygen on Electrical Properties of Amorphous Oxide Semiconductor, In-Ga-Zn-O. ECS J. Solid State Sci. Technol..

[65] Noh H.-K., Park J.-S., Chang K.J. (2013). Effect of hydrogen incorporation on the negative bias illumination stress instability in amorphous In-Ga-Zn-O thinfilm-transistors. J. Appl. Phys..

[66] Nahm H.-H., Park C.H., Kim Y.-S. (2014). Bistability of Hydrogen in ZnO: Origin of Doping Limit and Persistent Photoconductivity. Sci. Rep..

[67] Pan W., Wang Y., Wang Y., Xia Z., Yeung F.S.Y., Wong M., Kwok H.S., Wang X., Zhang S., Lu L. (2023). Multiple effects of hydrogen on InGaZnO thin-film transistor and the hydrogenation-resistibility enhancement. J. Alloys Compd..

[68] Kang Y., Ahn B.D., Song J.H., Mo Y.G., Nahm H., Han S., Jeong J.K. (2015). Hydrogen Bistability as the Origin of Photo-Bias-Thermal Instabilities in Amorphous Oxide Semiconductors. Adv. Electron. Mater..

[69] Miyase T., Watanabe K., Sakaguchi I., Ohashi N., Domen K., Nomura K., Hiramatsu H., Kumomi H., Hosono H., Kamiya T. (2014). Roles of Hydrogen in Amorphous Oxide Semiconductor In-Ga-Zn-O: Comparison of Conventional and Ultra-High-Vacuum Sputtering. ECS J. Solid State Sci. Technol..

[70] Kamiya T., Hosono H. (2013). (Invited) Roles of Hydrogen in Amorphous Oxide Semiconductor. ECS Trans..

[71] Chen T.-C., Chang T.-C., Tsai C.-T., Hsieh T.-Y., Chen S.-C., Lin C.-S., Hung M.-C., Tu C.-H., Chang J.-J., Chen P.-L. (2010). Behaviors of InGaZnO thin film transistor under illuminated positive gate-bias stress. Appl. Phys. Lett..

[72] Toledo P., Hernandez Luna I.S., Hernandez-Cuevas F., Hernandez-Como N. (2023). Electrical instabilities of a-IGZO TFTs under different conditions of bias and illumination stress. Microelectron. Reliab..

[73] Chowdhury M.D.H., Migliorato P., Jang J. (2011). Time-temperature dependence of positive gate bias stress and recovery in amorphous indium-gallium-zinc-oxide thin-film-transistors. Appl. Phys. Lett..

[74] Chen W.-T., Lo S.-Y., Kao S.-C., Zan H.-W., Tsai C.-C., Lin J.-H., Fang C.-H., Lee C.-C. (2011). Oxygen-Dependent Instability and Annealing/Passivation Effects in Amorphous In–Ga–Zn–O Thin-Film Transistors. IEEE Electron Device Lett..

[75] Aslam M., Chuang M.-H., Chang S.-W., Chen Y.-H., Lee Y.-J., Li Y. (2024). Temperature-Dependent Hydrogen Modulations of Ultra-Scaled a-IGZO Thin Film Transistor Under Gate Bias Stress. IEEE Open J. Nanotechnol..

[76] Tai Y.-H., Liu H.-W., Chan P.-C., Chiu S.-L. (2018). Degradation of a-IGZO Thin-Film Transistors Under Negative Bias and Illumination Stress in the Time Span of a Few Seconds. IEEE Electron Device Lett..

[77] Li S., Wang M., Zhang D., Wang H., Shan Q. (2019). A Unified Degradation Model of a-InGaZnO TFTs Under Negative Gate Bias with or Without an Illumination. IEEE J. Electron Devices Soc..

[78] Mativenga M., Haque F., Billah M.M., Um J.G. (2021). Origin of light instability in amorphous IGZO thin-film transistors and its suppression. Sci. Rep..

[79] Oh H., Yoon S.-M., Ryu M.K., Hwang C.-S., Yang S., Park S.-H.K. (2010). Photon-accelerated negative bias instability involving subgap states creation in amorphous In–Ga–Zn–O thin film transistor. Appl. Phys. Lett..

[80] Kim J., Oh B.S., Piao M., Joo M.-K., Jang H.-K., Ahn S.-E., Kim G.-T. (2014). Effects of low-temperature (120 °C) annealing on the carrier concentration and trap density in amorphous indium gallium zinc oxide thin film transistors. J. Appl. Phys..

[81] Jallorina M.P.A., Bermundo J.P.S., Fujii M.N., Ishikawa Y., Uraoka Y. (2018). Significant mobility improvement of amorphous In-Ga-Zn-O thin-film transistors annealed in a low temperature wet ambient environment. Appl. Phys. Lett..

[82] Kikuchi Y., Nomura K., Yanagi H., Kamiya T., Hirano M., Hosono H. (2010). Device characteristics improvement of a-In–Ga–Zn–O TFTs by low-temperature annealing. Thin Solid Film..

[83] Hanyu Y., Domen K., Nomura K., Hiramatsu H., Kumomi H., Hosono H., Kamiya T. (2013). Hydrogen passivation of electron trap in amorphous In-Ga-Zn-O thin-film transistors. Appl. Phys. Lett..

[84] Shin H.S., Ahn B.D., Rim Y.S., Kim H.J. (2011). Annealing temperature dependence on the positive bias stability of IGZO thin-film transistors. J. Inf. Disp..

[85] Choi S.-H., Lim M.-H., Jung W.-S., Park J.-H. (2014). Impacts of the Thermal Recovery Process on In–Ga–Zn–O (IGZO) TFTs. IEEE Electron Device Lett..

[86] Park S., Bang S., Lee S., Park J., Ko Y., Jeon H. (2011). The Effect of Annealing Ambient on the Characteristics of an Indium–Gallium–Zinc Oxide Thin Film Transistor. J. Nanosci. Nanotech..

[87] Huang X.D., Song J.Q., Lai P.T. (2016). Improved Performance of Scaled-Down α-InGaZnO Thin-Film Transistor by Ar Plasma Treatment. IEEE Electron Device Lett..

[88] Huang X.D., Ma Y., Song J.Q., Lai P.T., Tang W.M. (2018). Effects of Metal-Hydroxyl and InO_x_ Defects on Performance of InGaZnO Thin-Film Transistor. IEEE Trans. Electron Devices.

[89] Mudgal T., Walsh N., Manley R.G., Hirschman K.D. (2014). Impact of Annealing on Contact Formation and Stability of IGZO TFTs. ECS Trans..

[90] Ide K., Kikuchi Y., Nomura K., Kamiya T., Hosono H. (2012). Effects of low-temperature ozone annealing on operation characteristics of amorphous In–Ga–Zn–O thin-film transistors. Thin Solid Film..

[91] Peng C., Yang S., Pan C., Li X., Zhang J. (2020). Effect of Two-Step Annealing on High Stability of a-IGZO Thin-Film Transistor. IEEE Trans. Electron Devices.

[92] Jeon J.K., Um J.G., Lee S., Jang J. (2017). Control of O-H bonds at a-IGZO/SiO_2_ interface by long time thermal annealing for highly stable oxide TFT. AIP Adv..

[93] Pi T., Xiao D., Yang H., He G., Wu X., Liu W., Zhang D.W., Ding S.-J. (2022). High-Performance a-IGZO TFT Fabricated with Ultralow Thermal Budget via Microwave Annealing. IEEE Trans. Electron Devices.

[94] Lee J.-Y., Tarsoly G., Choi S.-G., Ryu H.-G., Kim S.-J. (2021). Influences of Oxygen Plasma Posttreatment on Electrical Characteristics of Amorphous Indium–Gallium–Zinc–Oxide Thin-Film Transistors. Phys. Status Solidi A.

[95] Abliz A. (2020). Effects of hydrogen plasma treatment on the electrical performances and reliability of InGaZnO thin-film transistors. J. Alloys Compd..

[96] Kim H., Han C., Kim D., Choi B. (2023). Electrical Performance and Reliability Enhancement of a-IGZO TFTs via Post-N_2_O Plasma Optimization. IEEE Trans. Electron Devices.

[97] Wang C., Peng C., Wen P., Xu M., Chen L., Li X., Zhang J. (2023). Improvement of Performance of Back Channel Etching InGaZnO Thin-Film Transistors by CF_4_ Plasma Treatment. IEEE Trans. Electron Devices.

[98] Huang X.D., Song J.Q., Lai P.T. (2017). Improved Stability of α-InGaZnO Thin-Film Transistor under Positive Gate Bias Stress by Using Fluorine Plasma Treatment. IEEE Electron Device Lett..

[99] Huo Y., Liu L., Liu G., Wang Z., Chang T.-L. (2021). 54-4: A 142-in. IGZO-TFT Glass-Substrate AM MiniLED Tiled Display with External Compensation and Multilayer Demura Algorithm. SID Symp. Dig. Tech. Pap..

[100] Sasaki T., Hakoi H., Hashimoto J., Ni M., Otsubo M., Sato T., Shimada S., Minoura K. (2020). 59-3: A Novel Transflective 31.5-inch IGZO-TFT LCD with a Twisted-VA Mode. SID Symp. Dig. Tech. Pap..

[101] Fan J., Lee C.Y., Chen S., Gang L.M., Jun Z.L., Yang S., Cai L.M., Fei X.H., Nian L., Shi J. (2019). 32-4: High transparent Active matrix Mini-LED Full Color Display with IGZO TFT Backplane. SID Symp. Dig. Tech. Pap..

[102] Chen W., Huang J.-J., Chen Y., Qian Y., Ruan S., Su C.-Y., Tseng C.-Y. (2021). P-1.3: Development of an 11.6-inch 144 Hz LCD Utilizing an IGZO TFT Backplane. SID Symp. Dig. Tech. Pap..

[103] Nakano F., Nakamura W., Hara Y., Ueda S., Minoura K., Chen W.T. (2023). 88-3: Late-News Paper: IGZO Backplane for Full-color Electrophoretic Display. SID Symp. Dig. Tech. Pap..

[104] Sun Y., Fan J., Liu M., Zhang L., Jiang B., Zhang M., Zhang X. (2020). Highly transparent, ultra-thin flexible, full-color mini-LED display with indium–gallium–zinc oxide thin-film transistor substrate. J. Soc. Info. Disp..

[105] Nakajima Y., Nakata M., Takei T., Fukagawa H., Motomura G., Tsuji H., Shimizu T., Fujisaki Y., Kurita T., Yamamoto T. (2014). Development of 8-in. oxide-TFT-driven flexible AMOLED display using high-performance red phosphorescent OLED. J. Soc. Info. Disp..

[106] Oota M., Hodo R., Ikeda T., Yamazaki S., Ando Y., Tsuda K., Koshida T., Oshita S., Suzuki A., Fukushima K. (2019). 3D-Stacked CAAC-In-Ga-Zn Oxide FETs with Gate Length of 72 nm. Proceedings of the 2019 IEEE International Electron Devices Meeting (IEDM).

[107] Belmonte A., Oh H., Rassoul N., Donadio G.L., Mitard J., Dekkers H., Delhougne R., Subhechha S., Chasin A., Van Setten M.J. (2020). Capacitor-less, Long-Retention (>400 s) DRAM Cell Paving the Way towards Low-Power and High-Density Monolithic 3D DRAM. Proceedings of the 2020 IEEE International Electron Devices Meeting (IEDM).

[108] Duan X., Huang K., Feng J., Niu J., Qin H., Yin S., Jiao G., Leonelli D., Zhao X., Wang Z. (2022). Novel Vertical Channel-All-Around (CAA) In-Ga-Zn-O FET for 2T0C-DRAM With High Density Beyond 4F^2^ by Monolithic Stacking. IEEE Trans. Electron Devices.

[109] Belmonte A., Oh H., Subhechha S., Rassoul N., Hody H., Dekkers H., Delhougne R., Ricotti L., Banerjee K., Chasin A. (2021). Tailoring IGZO-TFT architecture for capacitorless DRAM, demonstrating >10^3^ s retention, >10^11^ cycles endurance and L_g_ scalability down to 14 nm. Proceedings of the 2021 IEEE International Electron Devices Meeting (IEDM).

[110] Huang K., Duan X., Feng J., Sun Y., Lu C., Chen C., Jiao G., Lin X., Shao J., Yin S. (2022). Vertical Channel-All-Around (CAA) IGZO FET under 50 nm CD with High Read Current of 32.8 μA/μm (Vth + 1 V), Well-performed Thermal Stability up to 120 °C; for Low Latency, High-density 2T0C 3D DRAM Application. Proceedings of the 2022 IEEE Symposium on VLSI Technology and Circuits (VLSI Technology and Circuits).

[111] Lu W., Zhu Z., Chen K., Liu M., Kang B.-M., Duan X., Niu J., Liao F., Dan W., Wu X.-S. (2022). First Demonstration of Dual-Gate IGZO 2T0C DRAM with Novel Read Operation, One Bit Line in Single Cell, I_ON_ = 1500 μA/μm@V_DS_ = 1 V and Retention Time > 300 s. Proceedings of the 2022 International Electron Devices Meeting (IEDM).

[112] Hu Q., Li Q., Zhu S., Gu C., Liu S., Huang R., Wu Y. (2022). Optimized IGZO FETs for Capacitorless DRAM with Retention of 10 ks at RT and 7 ks at 85 °C at Zero V_hold_ with Sub-10 ns Speed and 3-bit Operation. Proceedings of the 2022 International Electron Devices Meeting (IEDM).

[113] Chen C., Duan X., Yang G., Lu C., Geng D., Li L., Liu M. (2022). Inter-Layer Dielectric Engineering for Monolithic Stacking 4F^2^-2T0C DRAM with Channel-All-Around (CAA) IGZO FET to Achieve Good Reliability (>10^4^ s Bias Stress, >10^12^ Cycles Endurance). Proceedings of the 2022 International Electron Devices Meeting (IEDM).

[114] Subhechha S., Rassoul N., Belmonte A., Hody H., Dekkers H., Van Setten M.J., Chasin A., Sharifi S.H., Sutar S., Magnarin L. (2022). Ultra-low Leakage IGZO-TFTs with Raised Source/Drain for V_t_ > 0 V and I_on_ > 30 µA/µm. Proceedings of the 2022 IEEE Symposium on VLSI Technology and Circuits (VLSI Technology and Circuits).

[115] Zhang Y.-Y., An S., Zheng Y., Lai J., Seo J.-H., Lee K.H., Kim M. (2022). Releasable AlGaN/GaN 2D Electron Gas Heterostructure Membranes for Flexible Wide-Bandgap Electronics. Adv. Electron. Mater..

[116] Gong J., Kim D., Jang H., Alema F., Wang Q., Zhou J., Li Y., Ng T.K., Qiu S., Liu Y. (2024). Characteristics of grafted monocrystalline Si/*β*-Ga2O3 *p*–*n* heterojunction. Appl. Phys. Lett..

[117] Gong J., Zhou J., Wang P., Kim T., Lu K., Min S., Singh R., Sheikhi M., Abbasi H.N., Vincent D. (2023). Synthesis and Characteristics of Transferrable Single-Crystalline AlN Nanomembranes. Adv. Electron. Mater..

[118] Tsao J.Y., Chowdhury S., Hollis M.A., Jena D., Johnson N.M., Jones K.A., Kaplar R.J., Rajan S., Van De Walle C.G., Bellotti E. (2018). Ultrawide-Bandgap Semiconductors: Research Opportunities and Challenges. Adv. Electron. Mater..

[119] Pensl G., Choyke W.J. (1993). Electrical and optical characterization of SiC. Phys. B Condens. Matter.

[120] Mourya S., Jaiswal J., Malik G., Kumar B., Chandra R. (2018). Structural and optical characteristics of in-situ sputtered highly oriented 15R-SiC thin films on different substrates. J. Appl. Phys..

[121] Park C.H., Cheong B.-H., Lee K.-H., Chang K.J. (1994). Structural and electronic properties of cubic, 2H, 4H, and 6H SiC. Phys. Rev. B.

[122] Persson C., Lindefelt U., Sernelius B.E. (1999). Band gap narrowing in *n* -type and *p* -type 3C-, 2H-, 4H-, 6H-SiC, and Si. J. Appl. Phys..

[123] Ching W.Y., Xu Y.-N., Rulis P., Ouyang L. (2006). The electronic structure and spectroscopic properties of 3C, 2H, 4H, 6H, 15R and 21R polymorphs of SiC. Mater. Sci. Eng. A.

[124] Wu R., Zhou K., Yue C.Y., Wei J., Pan Y. (2015). Recent progress in synthesis, properties and potential applications of SiC nanomaterials. Prog. Mater. Sci..

[125] Heine V., Cheng C., Needs R.J. (1991). The Preference of Silicon Carbide for Growth in the Metastable Cubic Form. J. Am. Ceram. Soc..

[126] Sik Yoo W., Matsunami H. (1991). Solid-State Phase Transformation in Cubic Silicon Carbide. Jpn. J. Appl. Phys..

[127] Boulle A., Dompoint D., Galben-Sandulache I., Chaussende D. (2013). Polytypic transformations in SiC: Diffuse x-ray scattering and Monte Carlo simulations. Phys. Rev. B.

[128] Ramakers S., Marusczyk A., Amsler M., Eckl T., Mrovec M., Hammerschmidt T., Drautz R. (2022). Effects of thermal, elastic, and surface properties on the stability of SiC polytypes. Phys. Rev. B.

[129] Peivaste I., Alahyarizadeh G., Minuchehr A., Aghaie M. (2018). Comparative study on mechanical properties of three different SiC polytypes (3C, 4H and 6H) under high pressure: First-principle calculations. Vacuum.

[130] Zorman C.A., Parro R.J. (2008). Micro- and nanomechanical structures for silicon carbide MEMS and NEMS. Phys. Status Solidi B.

[131] Contreras S., Konczewicz L., Arvinte R., Peyre H., Chassagne T., Zielinski M., Juillaguet S. (2017). Electrical transport properties of p-type 4H-SiC: Electrical transport properties of p-type 4H-SiC. Phys. Status Solidi A.

[132] Liang G., Qian H., Su Y., Shi L., Li Q., Liu Y. (2023). Review of solution growth techniques for 4H-SiC single crystal. China Foundry.

[133] Son N.T., Stenberg P., Jokubavicius V., Abe H., Ohshima T., Ul Hassan J., Ivanov I.G. (2019). Energy levels and charge state control of the carbon antisite-vacancy defect in 4H-SiC. Appl. Phys. Lett..

[134] Csóré A., Von Bardeleben H.J., Cantin J.L., Gali A. (2017). Characterization and formation of NV centers in 3C, 4H, and 6H SiC: An ab initio study. Phys. Rev. B.

[135] Yan X., Li P., Kang L., Wei S.-H., Huang B. (2020). First-principles study of electronic and diffusion properties of intrinsic defects in 4H-SiC. J. Appl. Phys..

[136] Dalibor T., Pensl G., Matsunami H., Kimoto T., Choyke W.J., Schöner A., Nordell N. (1997). Deep Defect Centers in Silicon Carbide Monitored with Deep Level Transient Spectroscopy. Phys. Stat. Sol. A.

[137] Hemmingsson C., Son N.T., Kordina O., Bergman J.P., Janzén E., Lindström J.L., Savage S., Nordell N. (1997). Deep level defects in electron-irradiated 4H SiC epitaxial layers. J. Appl. Phys..

[138] Klein P.B., Shanabrook B.V., Huh S.W., Polyakov A.Y., Skowronski M., Sumakeris J.J., O’Loughlin M.J. (2006). Lifetime-limiting defects in n− 4H-SiC epilayers. Appl. Phys. Lett..

[139] Danno K., Nakamura D., Kimoto T. (2007). Investigation of carrier lifetime in 4H-SiC epilayers and lifetime control by electron irradiation. Appl. Phys. Lett..

[140] Danno K., Kimoto T. (2006). Investigation of deep levels in n-type 4H-SiC epilayers irradiated with low-energy electrons. J. Appl. Phys..

[141] Sasaki S., Kawahara K., Feng G., Alfieri G., Kimoto T. (2011). Major deep levels with the same microstructures observed in n-type 4H–SiC and 6H–SiC. J. Appl. Phys..

[142] Zhang J., Storasta L., Bergman J.P., Son N.T., Janzén E. (2003). Electrically active defects in *n*-type 4H–silicon carbide grown in a vertical hot-wall reactor. J. Appl. Phys..

[143] Storasta L., Bergman J.P., Janzén E., Henry A., Lu J. (2004). Deep levels created by low energy electron irradiation in 4H-SiC. J. Appl. Phys..

[144] Capan I., Brodar T., Pastuović Ž., Siegele R., Ohshima T., Sato S., Makino T., Snoj L., Radulović V., Coutinho J. (2018). Double negatively charged carbon vacancy at the h- and k-sites in 4H-SiC: Combined Laplace-DLTS and DFT study. J. Appl. Phys..

[145] Hornos T., Gali A., Svensson B.G. (2011). Large-Scale Electronic Structure Calculations of Vacancies in 4H-SiC Using the Heyd-Scuseria-Ernzerhof Screened Hybrid Density Functional. MSF.

[146] Son N.T., Trinh X.T., Løvlie L.S., Svensson B.G., Kawahara K., Suda J., Kimoto T., Umeda T., Isoya J., Makino T. (2012). Negative-*U* System of Carbon Vacancy in 4*H*-SiC. Phys. Rev. Lett..

[147] Hemmingsson C.G., Son N.T., Ellison A., Zhang J., Janzén E. (1998). Negative-*U* centers in 4*H* silicon carbide. Phys. Rev. B.

[148] Coutinho J., Torres V.J.B., Demmouche K., Öberg S. (2017). Theory of the carbon vacancy in 4*H*-SiC: Crystal field and pseudo-Jahn-Teller effects. Phys. Rev. B.

[149] Bockstedte M., Marini A., Pankratov O., Rubio A. (2010). Many-Body Effects in the Excitation Spectrum of a Defect in SiC. Phys. Rev. Lett..

[150] Huang Y., Wang R., Zhang Y., Yang D., Pi X. (2022). Compensation of *p* -type doping in Al-doped 4H-SiC. J. Appl. Phys..

[151] Bathen M.E., Coutinho J., Ayedh H.M., Ul Hassan J., Farkas I., Öberg S., Frodason Y.K., Svensson B.G., Vines L. (2019). Anisotropic and plane-selective migration of the carbon vacancy in SiC: Theory and experiment. Phys. Rev. B.

[152] Coutinho J., Gouveia J.D., Makino T., Ohshima T., Pastuović Ž., Bakrač L., Brodar T., Capan I. (2021). *M* center in 4*H*-SiC is a carbon self-interstitial. Phys. Rev. B.

[153] Storasta L., Tsuchida H., Miyazawa T., Ohshima T. (2008). Enhanced annealing of the Z1∕2 defect in 4H–SiC epilayers. J. Appl. Phys..

[154] Hiyoshi T., Kimoto T. (2009). Reduction of Deep Levels and Improvement of Carrier Lifetime in n-Type 4H-SiC by Thermal Oxidation. Appl. Phys. Express.

[155] Kawahara K., Suda J., Kimoto T. (2012). Analytical model for reduction of deep levels in SiC by thermal oxidation. J. Appl. Phys..

[156] Bathen M.E., Karsthof R., Galeckas A., Kumar P., Kuznetsov A.Y., Grossner U., Vines L. (2024). Impact of carbon injection in 4H-SiC on defect formation and minority carrier lifetime. Mater. Sci. Semicond. Process..

[157] Ayedh H.M., Nipoti R., Hallén A., Svensson B.G. (2015). Elimination of carbon vacancies in 4H-SiC employing thermodynamic equilibrium conditions at moderate temperatures. Appl. Phys. Lett..

[158] Kobayashi T., Harada K., Kumagai Y., Oba F., Matsushita Y. (2019). Native point defects and carbon clusters in 4H-SiC: A hybrid functional study. J. Appl. Phys..

[159] Torpo L., Marlo M., Staab T.E.M., Nieminen R.M. (2001). Comprehensive ab initio study of properties of monovacancies and antisites in 4H-SiC. J. Phys. Condens. Matter.

[160] Wang X., Zhao J., Xu Z., Djurabekova F., Rommel M., Song Y., Fang F. (2020). Density functional theory calculation of the properties of carbon vacancy defects in silicon carbide. Nanotechnol. Precis. Eng..

[161] Cochrane C.J., Lenahan P.M., Lelis A.J. (2012). Identification of a silicon vacancy as an important defect in 4H SiC metal oxide semiconducting field effect transistor using spin dependent recombination. Appl. Phys. Lett..

[162] Bathen M.E., Galeckas A., Coutinho J., Vines L. (2020). Influence of hydrogen implantation on emission from the silicon vacancy in 4H-SiC. J. Appl. Phys..

[163] Janzén E., Gali A., Carlsson P., Gällström A., Magnusson B., Son N.T. (2009). The silicon vacancy in SiC. Phys. B Condens. Matter.

[164] Kobayashi T., Shimura T., Watanabe H. (2023). Oxygen-vacancy defect in 4H-SiC as a near-infrared emitter: An ab initio study. J. Appl. Phys..

[165] Lukin D.M., Dory C., Guidry M.A., Yang K.Y., Mishra S.D., Trivedi R., Radulaski M., Sun S., Vercruysse D., Ahn G.H. (2020). 4H-silicon-carbide-on-insulator for integrated quantum and nonlinear photonics. Nat. Photonics.

[166] Carter S.G., Soykal Ö.O., Dev P., Economou S.E., Glaser E.R. (2015). Spin coherence and echo modulation of the silicon vacancy in 4*H*-SiC at room temperature. Phys. Rev. B.

[167] Baranov P.G., Bundakova A.P., Soltamova A.A., Orlinskii S.B., Borovykh I.V., Zondervan R., Verberk R., Schmidt J. (2011). Silicon vacancy in SiC as a promising quantum system for single-defect and single-photon spectroscopy. Phys. Rev. B.

[168] Davidsson J., Babar R., Shafizadeh D., Ivanov I.G., Ivády V., Armiento R., Abrikosov I.A. (2022). Exhaustive characterization of modified Si vacancies in 4H-SiC. Nanophotonics.

[169] Soykal Ö.O., Dev P., Economou S.E. (2016). Silicon vacancy center in 4*H*-SiC: Electronic structure and spin-photon interfaces. Phys. Rev. B.

[170] Nagy R., Niethammer M., Widmann M., Chen Y.-C., Udvarhelyi P., Bonato C., Hassan J.U., Karhu R., Ivanov I.G., Son N.T. (2019). High-fidelity spin and optical control of single silicon-vacancy centres in silicon carbide. Nat. Commun..

[171] Widmann M., Lee S.-Y., Rendler T., Son N.T., Fedder H., Paik S., Yang L.-P., Zhao N., Yang S., Booker I. (2015). Coherent control of single spins in silicon carbide at room temperature. Nat. Mater..

[172] Wang J.-F., Yan F.-F., Li Q., Liu Z.-H., Cui J.-M., Liu Z.-D., Gali A., Xu J.-S., Li C.-F., Guo G.-C. (2021). Robust coherent control of solid-state spin qubits using anti-Stokes excitation. Nat. Commun..

[173] Simin D., Soltamov V.A., Poshakinskiy A.V., Anisimov A.N., Babunts R.A., Tolmachev D.O., Mokhov E.N., Trupke M., Tarasenko S.A., Sperlich A. (2016). All-Optical dc Nanotesla Magnetometry Using Silicon Vacancy Fine Structure in Isotopically Purified Silicon Carbide. Phys. Rev. X.

[174] Wang J.-F., Liu L., Liu X.-D., Li Q., Cui J.-M., Zhou D.-F., Zhou J.-Y., Wei Y., Xu H.-A., Xu W. (2023). Magnetic detection under high pressures using designed silicon vacancy centres in silicon carbide. Nat. Mater..

[175] Lohrmann A., Johnson B.C., McCallum J.C., Castelletto S. (2017). A review on single photon sources in silicon carbide. Rep. Prog. Phys..

[176] Capan I., Brodar T., Bernat R., Pastuović Ž., Makino T., Ohshima T., Gouveia J.D., Coutinho J. (2021). M-center in 4H-SiC: Isothermal DLTS and first principles modeling studies. J. Appl. Phys..

[177] Li P., Udvarhelyi P., Li S., Huang B., Gali A. (2023). Carbon cluster emitters in silicon carbide. Phys. Rev. B.

[178] Knežević T., Hadžipašić A., Ohshima T., Makino T., Capan I. (2022). M-center in low-energy electron irradiated 4*H*-SiC. Appl. Phys. Lett..

[179] Karsthof R., Etzelmüller Bathen M., Kuznetsov A., Vines L. (2022). Formation of carbon interstitial-related defect levels by thermal injection of carbon into *n*-type 4*H*-SiC. J. Appl. Phys..

[180] Knežević T., Brodar T., Radulović V., Snoj L., Makino T., Capan I. (2022). Distinguishing the EH_1_ and S_1_ defects in n-type 4H-SiC by Laplace DLTS. Appl. Phys. Express.

[181] Alfieri G., Mihaila A. (2020). Isothermal annealing study of the EH1 and EH3 levels in n-type 4H-SiC. J. Phys. Condens. Matter.

[182] Liao T., Roma G., Wang J. (2009). First-principles study of neutral silicon interstitials in 3C- and 4H-SiC. Philos. Mag..

[183] Coutinho J. (2021). Theory of the Thermal Stability of Silicon Vacancies and Interstitials in 4H–SiC. Crystals.

[184] Nakane H., Kato M., Ohkouchi Y., Trinh X.T., Ivanov I.G., Ohshima T., Son N.T. (2021). Deep levels related to the carbon antisite–vacancy pair in 4H-SiC. J. Appl. Phys..

[185] Son N.T., Hai P.N., Janzén E. (2001). Silicon Antisite in 4*H* SiC. Phys. Rev. Lett..

[186] Wang R., Huang Y., Yang D., Pi X. (2023). Impurities and defects in 4H silicon carbide. Appl. Phys. Lett..

[187] Ivanov I.G., Magnusson B., Janzén E. (2003). Analysis of the sharp donor-acceptor pair luminescence in 4*H*-SiC doped with nitrogen and aluminum. Phys. Rev. B.

[188] Evwaraye A.O., Smith S.R., Mitchel W.C. (1996). Shallow and deep levels in *n* -type 4H-SiC. J. Appl. Phys..

[189] Lebedev A.A. (1999). Deep level centers in silicon carbide: A review. Semiconductors.

[190] Gerstmann U., Rauls E., Frauenheim T., Overhof H. (2003). Formation and annealing of nitrogen-related complexes in SiC. Phys. Rev. B.

[191] Liu X., Zhang J., Xu B., Lu Y., Zhang Y., Wang R., Yang D., Pi X. (2022). Deformation of 4H-SiC: The role of dopants. Appl. Phys. Lett..

[192] Wang R., Liu X., Li J., Luo H., Yang G., Yang D., Pi X. (2022). Effect of nitrogen doping on the dislocation behaviors of 4H-SiC. Proceedings of the 2022 6th IEEE Electron Devices Technology & Manufacturing Conference (EDTM).

[193] Darmody C., Goldsman N. (2019). Incomplete ionization in aluminum-doped 4H-silicon carbide. J. Appl. Phys..

[194] Huang Y., Wang R., Qian Y., Zhang Y., Yang D., Pi X. (2022). Theoretical study on the improvement of the doping efficiency of Al in 4H-SiC by co-doping group-IVB elements. Chin. Phys. B.

[195] Forsberg U., Danielsson Ö., Henry A., Linnarsson M.K., Janzén E. (2003). Aluminum doping of epitaxial silicon carbide. J. Cryst. Growth.

[196] Sridhara S.G., Clemen L.L., Devaty R.P., Choyke W.J., Larkin D.J., Kong H.S., Troffer T., Pensl G. (1998). Photoluminescence and transport studies of boron in 4H SiC. J. Appl. Phys..

[197] Torres V.J.B., Capan I., Coutinho J. (2022). Theory of shallow and deep boron defects in 4H-SiC. Phys. Rev. B.

[198] Ghezellou M., Kumar P., Bathen M.E., Karsthof R., Sveinbjörnsson E.Ö., Grossner U., Bergman J.P., Vines L., Ul-Hassan J. (2023). The role of boron related defects in limiting charge carrier lifetime in 4H–SiC epitaxial layers. APL Mater..

[199] Beyer F.C., Hemmingsson C.G., Leone S., Lin Y.-C., Gällström A., Henry A., Janzén E. (2011). Deep levels in iron doped n- and p-type 4H-SiC. J. Appl. Phys..

[200] Song H.K., Kwon S.Y., Seo H.S., Moon J.H., Yim J.H., Lee J.H., Kim H.J., Jeong J.K. (2006). Homoepitaxial growth and electrical characterization of iron-doped semi-insulating 4H-SiC epilayer. Appl. Phys. Lett..

[201] Lu X., Zhao T., Guo X., Chen M., Ren J., La P. (2019). Electronic structures and optical properties of Ni-doped 4H-SiC: Dispersion-corrected density functional theory investigations. Mater. Res. Express.

[202] Lin L., Huang J., Yu W., Tao H., Zhu L., Wang P., Zhang Z., Zhang J. (2018). Electronic structures and magnetic properties of (Ni,Al) co-doped 4H-SiC: A first-principles study. Comput. Mater. Sci..

[203] Mitchel W.C., Mitchell W.D., Landis G., Smith H.E., Lee W., Zvanut M.E. (2007). Vanadium donor and acceptor levels in semi-insulating 4H- and 6H-SiC. J. Appl. Phys..

[204] Huang Y., Wang R., Zhang N., Zhang Y., Yang D., Pi X. (2022). Effect of hydrogen on the unintentional doping of 4H silicon carbide. J. Appl. Phys..

[205] Shen X., Pantelides S.T. (2011). Identification of a major cause of endemically poor mobilities in SiC/SiO_2_ structures. Appl. Phys. Lett..

[206] Kaneko T., Tajima N., Yamasaki T., Nara J., Schimizu T., Kato K., Ohno T. (2018). Hybrid density functional analysis of distribution of carbon-related defect levels at 4H-SiC(0001)/SiO_2_ interface. Appl. Phys. Express.

[207] Li W., Zhao J., Wang D. (2015). An amorphous SiO_2_/4H-SiC(0001) interface: Band offsets and accurate charge transition levels of typical defects. Solid State Commun..

[208] Shen X., Pantelides S.T. (2012). Oxidation-Induced Epilayer Carbon Di-Interstitials as a Major Cause of Endemically Poor Mobilities in 4H-SiC/SiO_2_ Structures. MSF.

[209] Kagoyama Y., Okamoto M., Yamasaki T., Tajima N., Nara J., Ohno T., Yano H., Harada S., Umeda T. (2019). Anomalous carbon clusters in 4H-SiC/SiO_2_ interfaces. J. Appl. Phys..

[210] Dutta D., De D.S., Fan D., Roy S., Alfieri G., Camarda M., Amsler M., Lehmann J., Bartolf H., Goedecker S. (2019). Evidence for carbon clusters present near thermal gate oxides affecting the electronic band structure in SiC-MOSFET. Appl. Phys. Lett..

[211] Zhang Z., Wang Z., Guo Y., Robertson J. (2021). Carbon cluster formation and mobility degradation in 4H-SiC MOSFETs. Appl. Phys. Lett..

[212] Li W., Zhao J., Wang D. (2015). Structural and electronic properties of the transition layer at the SiO_2_/4H-SiC interface. AIP Adv..

[213] Johnson B.C., Woerle J., Haasmann D., Lew C.T.-K., Parker R.A., Knowles H., Pingault B., Atature M., Gali A., Dimitrijev S. (2019). Optically Active Defects at the SiC/SiO_2_ Interface. Phys. Rev. Appl..

[214] Wei S., Yin Z., Bai J., Xie W., Qin F., Su Y., Wang D. (2023). The initial oxidation of the 4H-SiC (0001) surface with C-related point defects: Insight by first-principles calculations. Appl. Surf. Sci..

[215] Afanas’ev V.V., Ciobanu F., Dimitrijev S., Pensl G., Stesmans A. (2005). SiC/SiO_2_ Interface States: Properties and Models. MSF.

[216] Shimizu T., Akiyama T., Ito T., Kageshima H., Uematsu M., Shiraishi K. (2021). Ab initio-based approach for the oxidation mechanisms at SiO_2_/4H-SiC interface: Interplay of dry and wet oxidants during interfacial reaction. Phys. Rev. Mater..

[217] Zhang Z., Guo Y., Robertson J. (2021). Mobility degradation in 4H-SiC MOSFETs and interfacial formation of carbon clusters. Solid State Electron..

[218] Afanas’ev V.V., Stesmans A., Harris C.I. (1998). Observation of Carbon Clusters at the 4H-SiC/SiO_2_ Interface. MSF.

[219] Jiang C., Morgan D., Szlufarska I. (2012). Carbon tri-interstitial defect: A model for the D_II_ center. Phys. Rev. B.

[220] Bathen M.E., Vines L. (2021). Manipulating Single-Photon Emission from Point Defects in Diamond and Silicon Carbide. Adv. Quantum Tech..

[221] Kawahara K., Suda J., Kimoto T. (2013). Deep levels generated by thermal oxidation in p-type 4H-SiC. J. Appl. Phys..

[222] Devynck F., Alkauskas A., Broqvist P., Pasquarello A., Caldas M., Studart N. Energy levels of candidate defects at SiC/SiO_2_ interfaces. Proceedings of the 29th International Conference on the Physics of Semiconductors.

[223] Castelletto S., Johnson B.C., Ivády V., Stavrias N., Umeda T., Gali A., Ohshima T. (2014). A silicon carbide room-temperature single-photon source. Nat. Mater.

[224] Rühl M., Bergmann L., Krieger M., Weber H.B. (2020). Stark Tuning of the Silicon Vacancy in Silicon Carbide. Nano Lett..

[225] Lingner T., Greulich-Weber S., Spaeth J.-M., Gerstmann U., Rauls E., Hajnal Z., Frauenheim T., Overhof H. (2001). Structure of the silicon vacancy in 6*H*-SiC after annealing identified as the carbon vacancy–carbon antisite pair. Phys. Rev. B.

[226] Lee E.M.Y., Yu A., De Pablo J.J., Galli G. (2021). Stability and molecular pathways to the formation of spin defects in silicon carbide. Nat Commun..

[227] Bockstedte M., Mattausch A., Pankratov O. (2003). Ab initio study of the migration of intrinsic defects in 3C-SiC. Phys. Rev. B.

[228] Umeda T., Son N.T., Isoya J., Janzén E., Ohshima T., Morishita N., Itoh H., Gali A., Bockstedte M. (2006). Identification of the Carbon Antisite-Vacancy Pair in 4*H*-SiC. Phys. Rev. Lett..

[229] Umeda T., Ishoya J., Ohshima T., Morishita N., Itoh H., Gali A. (2007). Identification of positively charged carbon antisite-vacancy pairs in 4H-SiC. Phys. Rev. B.

[230] Szász K., Ivády V., Abrikosov I.A., Janzén E., Bockstedte M., Gali A. (2015). Spin and photophysics of carbon-antisite vacancy defect in 4H silicon carbide: A potential quantum bit. Phys. Rev. B.

[231] Yi A., Wang C., Zhou L., Zhu Y., Zhang S., You T., Zhang J., Ou X. (2022). Silicon carbide for integrated photonics. Appl. Phys. Rev..

[232] Klein P.B. (2008). Carrier lifetime measurement in n− 4H-SiC epilayers. J. Appl. Phys..

[233] Kimoto T., Danno K., Suda J. (2008). Lifetime-killing defects in 4H-SiC epilayers and lifetime control by low-energy electron irradiation. Phys. Status Solidi B.

[234] Kimoto T., Niwa H., Okuda T., Saito E., Zhao Y., Asada S., Suda J. (2018). Carrier lifetime and breakdown phenomena in SiC power device material. J. Phys. D Appl. Phys..

[235] Saito E., Suda J., Kimoto T. (2016). Control of carrier lifetime of thick n-type 4H-SiC epilayers by high-temperature Ar annealing. Appl. Phys. Express.

[236] Kimoto T. (2015). Material science and device physics in SiC technology for high-voltage power devices. Jpn. J. Appl. Phys..

[237] Ayedh H.M., Hallén A., Svensson B.G. (2015). Elimination of carbon vacancies in 4H-SiC epi-layers by near-surface ion implantation: Influence of the ion species. J. Appl. Phys..

[238] Okamoto D., Yano H., Hatayama T., Fuyuki T. (2010). Removal of near-interface traps at SiO_2_/4H–SiC (0001) interfaces by phosphorus incorporation. Appl. Phys. Lett..

[239] Fiorenza P., Bongiorno C., Giannazzo F., Alessandrino M.S., Messina A., Saggio M., Roccaforte F. (2021). Interfacial electrical and chemical properties of deposited SiO_2_ layers in lateral implanted 4H-SiC MOSFETs subjected to different nitridations. Appl. Surf. Sci..

[240] Tachiki K., Kaneko M., Kimoto T. (2021). Mobility improvement of 4H-SiC (0001) MOSFETs by a three-step process of H_2_ etching, SiO_2_ deposition, and interface nitridation. Appl. Phys. Express.

[241] Lelis A.J., Green R., Habersat D.B., El M. (2015). Basic Mechanisms of Threshold-Voltage Instability and Implications for Reliability Testing of SiC MOSFETs. IEEE Trans. Electron Devices.

[242] Yano H., Kanafuji N., Osawa A., Hatayama T., Fuyuki T. (2015). Threshold Voltage Instability in 4H-SiC MOSFETs With Phosphorus-Doped and Nitrided Gate Oxides. IEEE Trans. Electron Devices.

[243] Karadavut O., Chaudhuri S.K., Kleppinger J.W., Nag R., Mandal K.C. (2022). Enhancement of radiation detection performance with reduction of EH_6/7_ deep levels in n-type 4H–SiC through thermal oxidation. Appl. Phys. Lett..

[244] Ichikawa S., Kawahara K., Suda J., Kimoto T. (2012). Carrier Recombination in n-Type 4H-SiC Epilayers with Long Carrier Lifetimes. Appl. Phys. Express.

[245] Okuda T., Miyazawa T., Tsuchida H., Kimoto T., Suda J. (2014). Enhancement of carrier lifetime in lightly Al-doped p-type 4H-SiC epitaxial layers by combination of thermal oxidation and hydrogen annealing. Appl. Phys. Express.

[246] Murata K., Tawara T., Yang A., Takanashi R., Miyazawa T., Tsuchida H. (2019). Wide-ranging control of carrier lifetimes in n-type 4H-SiC epilayer by intentional vanadium doping. J. Appl. Phys..

[247] Yoshioka H., Nakamura T., Kimoto T. (2012). Generation of very fast states by nitridation of the SiO_2_/SiC interface. J. Appl. Phys..

[248] Fujihira K., Tarui Y., Imaizumi M., Ohtsuka K., Takami T., Shiramizu T., Kawase K., Tanimura J., Ozeki T. (2005). Characteristics of 4H–SiC MOS interface annealed in N_2_O. Solid State Electron..

[249] Komatsu N., Ohmoto M., Uemoto M., Ono T. (2022). Density functional theory calculations for investigation of atomic structures of 4H-SiC/SiO_2_ interface after NO annealing. J. Appl. Phys..

[250] Akiyama T., Kageshima H., Shiraishi K. (2024). Reaction of NO molecule at 4H-SiC/SiO_2_ interface and its orientation dependence: A first-principles study. Jpn. J. Appl. Phys..

[251] Tachiki K., Kimoto T. (2021). Improvement of Both n- and p-Channel Mobilities in 4H-SiC MOSFETs by High-Temperature N₂ Annealing. IEEE Trans. Electron Devices.

[252] Chanthaphan A., Hosoi T., Shimura T., Watanabe H. (2015). Study of SiO_2_/4H-SiC interface nitridation by post-oxidation annealing in pure nitrogen gas. AIP Adv..

[253] Akiyama T., Shimizu T., Ito T., Kageshima H., Shiraishi K. (2022). Reaction of nitrous oxide and ammonia molecules at 4H-SiC/SiO_2_ interface: An ab initio study. Surf. Sci..

[254] Wang Z., Zhang Z., Shao C., Robertson J., Liu S., Guo Y. (2021). Defects and Passivation Mechanism of the Suboxide Layers at SiO₂/4H-SiC (0001) Interface: A First-Principles Calculation. IEEE Trans. Electron Devices.

[255] Shimizu T., Akiyama T., Nakamura K., Ito T., Kageshima H., Uematsu M., Shiraishi K. (2021). Reaction of NO molecule at 4H-SiC/SiO_2_ interface: An ab initio study for the effect of NO annealing after dry oxidation. Jpn. J. Appl. Phys..

[256] Wang M., Yang M., Liu W., Qi J., Yang S., Han C., Geng L., Hao Y. (2021). A Highly Efficient Annealing Process with Supercritical N_2_O at 120 °C for SiO_2_/4H–SiC Interface. IEEE Trans. Electron Devices.

[257] Sun Y., Yang C., Yin Z., Qin F., Wang D. (2019). Plasma passivation of near-interface oxide traps and voltage stability in SiC MOS capacitors. J. Appl. Phys..

[258] Fujimoto H., Kobayashi T., Sometani M., Okamoto M., Shimura T., Watanabe H. (2022). Degradation of NO-nitrided SiC MOS interfaces by excimer ultraviolet light irradiation. Appl. Phys. Express.

[259] Kobayashi T., Kimoto T. (2017). Carbon ejection from a SiO_2_/SiC(0001) interface by annealing in high-purity Ar. Appl. Phys. Lett..

[260] Kobayashi T., Suda J., Kimoto T. (2017). Reduction of interface state density in SiC (0001) MOS structures by post-oxidation Ar annealing at high temperature. AIP Adv..

[261] Fujimoto H., Kobayashi T., Shimura T., Watanabe H. (2023). Improvement of interface properties in SiC(0001) MOS structures by plasma nitridation of SiC surface followed by SiO_2_ deposition and CO_2_ annealing. Appl. Phys. Express.

[262] Puschkarsky K., Grasser T., Aichinger T., Gustin W., Reisinger H. (2019). Review on SiC MOSFETs High-Voltage Device Reliability Focusing on Threshold Voltage Instability. IEEE Trans. Electron Devices.

[263] Ettisserry D.P., Goldsman N., Akturk A., Lelis A.J. (2014). Structure, bonding, and passivation of single carbon-related oxide hole traps near 4H-SiC/SiO_2_ interfaces. J. Appl. Phys..

[264] Ettisserry D.P., Goldsman N., Lelis A.J. (2017). Role of Oxygen Vacancies in Short- and Long-Term Instability of Negative Bias-Temperature Stressed SiC MOSFETs. IEEE Trans. Electron Devices.

[265] Rozen J., Dhar S., Zvanut M.E., Williams J.R., Feldman L.C. (2009). Density of interface states, electron traps, and hole traps as a function of the nitrogen density in SiO_2_ on SiC. J. Appl. Phys..

[266] Noguchi M., Iwamatsu T., Amishiro H., Watanabe H., Kita K., Miura N. (2019). Improvement in the Channel Performance and NBTI of SiC-MOSFETs by Oxygen Doping. Proceedings of the 2019 IEEE International Electron Devices Meeting (IEDM).

[267] Lelis A.J., Habersat D., Green R., Ogunniyi A., Gurfinkel M., Suehle J., Goldsman N. (2008). Time Dependence of Bias-Stress-Induced SiC MOSFET Threshold-Voltage Instability Measurements. IEEE Trans. Electron Devices.

[268] Chen Z., Huang A.Q. (2024). Extreme high efficiency enabled by silicon carbide (SiC) power devices. Mater. Sci. Semicond. Process..

[269] Yuan X., Laird I., Walder S. (2021). Opportunities, Challenges, and Potential Solutions in the Application of Fast-Switching SiC Power Devices and Converters. IEEE Trans. Power Electron..

[270] Xun Q., Xun B., Li Z., Wang P., Cai Z. (2017). Application of SiC power electronic devices in secondary power source for aircraft. Renew. Sustain. Energy Rev..

[271] Adamowicz M., Szewczyk J. (2020). SiC-Based Power Electronic Traction Transformer (PETT) for 3 kV DC Rail Traction. Energies.

[272] Ding R., Dou Z., Qi Y., Mei W., Liu G. (2022). Analysis on characteristic of 3.3-kV full SiC device and railway traction converter design. IET Power Electron..

[273] Eguchi H. (2022). Technology trends of automotive semiconductors for CASE application. Proceedings of the 2022 International Power Electronics Conference (IPEC-Himeji 2022- ECCE Asia).

[274] Schefer H., Fauth L., Kopp T.H., Mallwitz R., Friebe J., Kurrat M. (2020). Discussion on Electric Power Supply Systems for All Electric Aircraft. IEEE Access.

[275] Barzkar A., Ghassemi M. (2022). Components of Electrical Power Systems in More and All-Electric Aircraft: A Review. IEEE Trans. Transp. Electrific..

[276] Kimoto T. (2022). High-voltage SiC power devices for improved energy efficiency. Proc. Jpn. Acad. Ser. B.

[277] Allca-Pekarovic A., Kollmeyer P.J., Mahvelatishamsabadi P., Mirfakhrai T., Naghshtabrizi P., Emadi A. (2020). Comparison of IGBT and SiC Inverter Loss for 400V and 800V DC Bus Electric Vehicle Drivetrains. Proceedings of the 2020 IEEE Energy Conversion Congress and Exposition (ECCE).

[278] Pradhan R., Keshmiri N., Emadi A. (2023). On-Board Chargers for High-Voltage Electric Vehicle Powertrains: Future Trends and Challenges. IEEE Open J. Power Electron..

[279] Kouchaki A., Nymand M. (2017). High efficiency three-phase power factor correction rectifier using SiC switches. Proceedings of the 2017 19th European Conference on Power Electronics and Applications (EPE’17 ECCE Europe).

[280] Zhu K., Bhalla A., Dodge J. (2021). Enabling 99.3% Efficiency in 3.6 kW Totem-Pole PFC Using New 750 V Gen 4 SiC FETs. IEEE Power Electron. Mag..

[281] Saha J., Kumar N., Panda S.K. (2023). A Futuristic Silicon-Carbide (SiC)-Based Electric-Vehicle Fast Charging/Discharging (FC/dC) Station. IEEE J. Emerg. Sel. Top. Power Electron..

[282] Li L., Yuan S., Amina K., Zhai P., Su Y., Lou R., Hao X., Shan H., Xue T., Liu H. (2022). Robust and fast response solar-blind UV photodetectors based on the transferable 4H-SiC free-standing nanowire arrays. Sens. Actuators A Phys..

[283] Mo J., Li J., Zhang Y., Romijn J., May A., Erlbacher T., Zhang G., Vollebregt S. (2023). A Highly Linear Temperature Sensor Operating up to 600 °C in a 4H-SiC CMOS Technology. IEEE Electron Device Lett..

[284] Soltau N., Wiesner E., Stumpf E., Idaka S., Hatori K. (2020). Electric-Energy Savings using 3.3 kV Full-SiC Power-Modules in Traction Applications. Proceedings of the 2020 Fifteenth International Conference on Ecological Vehicles and Renewable Energies (EVER).

[285] He J., Sangwongwanich A., Yang Y., Zhang K., Iannuzzo F. (2022). Design for Reliability of SiC-MOSFET-Based 1500-V PV Inverters with Variable Gate Resistance. IEEE Trans. Ind. Applicat..

[286] Chen Z., Rizi H.S., Xu W., Yu R., Huang A.Q. (2022). Hardware Design of a 150 kW/1500 V All-SiC Grid-forming Photovoltaic Synchronous Generator (PVSG). Proceedings of the 2022 IEEE Applied Power Electronics Conference and Exposition (APEC).

